# Ranking major and minor research misbehaviors: results from a survey among participants of four World Conferences on Research Integrity

**DOI:** 10.1186/s41073-016-0024-5

**Published:** 2016-11-21

**Authors:** Lex M. Bouter, Joeri Tijdink, Nils Axelsen, Brian C. Martinson, Gerben ter Riet

**Affiliations:** 1 Department of Epidemiology and Biostatistics, VU University Medical Center, Amsterdam, The Netherlands; 2 Department of Philosophy, Faculty of Humanities, Vrije Universiteit, Amsterdam, The Netherlands; 3 Department of Internal Medicine, VU University Medical Center, Amsterdam, The Netherlands; 4 Office of Research Integrity, Statens Serum Institut, Copenhagen, Denmark; 5 Department of Medicine, HealthPartners Institute and Minneapolis Veterans Affairs, Center for Chronic Disease Outcomes Research and University of Minnesota, Minneapolis, MN USA; 6 Department of General Practice, Academic Medical Center, University of Amsterdam, Amsterdam, The Netherlands

**Keywords:** Research integrity, Responsible conduct of research, Questionable research practices, Sloppy science, Research misconduct, Fabrication, Falsification, Plagiarism

## Abstract

**Background:**

Codes of conduct mainly focus on research misconduct that takes the form of fabrication, falsification, and plagiarism. However, at the aggregate level, lesser forms of research misbehavior may be more important due to their much higher prevalence. Little is known about what the most frequent research misbehaviors are and what their impact is if they occur.

**Methods:**

A survey was conducted among 1353 attendees of international research integrity conferences. They were asked to score 60 research misbehaviors according to their views on and perceptions of the frequency of occurrence, preventability, impact on truth (validity), and impact on trust between scientists on 5-point scales. We expressed the aggregate level impact as the product of frequency scores and truth, trust and preventability scores, respectively. We ranked misbehaviors based on mean scores. Additionally, relevant demographic and professional background information was collected from participants.

**Results:**

Response was 17% of those who were sent the invitational email and 33% of those who opened it. The rankings suggest that selective reporting, selective citing, and flaws in quality assurance and mentoring are viewed as the major problems of modern research. The “deadly sins” of fabrication and falsification ranked highest on the impact on truth but low to moderate on aggregate level impact on truth, due to their low estimated frequency. Plagiarism is thought to be common but to have little impact on truth although it ranked high on aggregate level impact on trust.

**Conclusions:**

We designed a comprehensive list of 60 major and minor research misbehaviors. Our respondents were much more concerned over sloppy science than about scientific fraud (FFP). In the fostering of responsible conduct of research, we recommend to develop interventions that actively discourage the high ranking misbehaviors from our study.

**Electronic supplementary material:**

The online version of this article (doi:10.1186/s41073-016-0024-5) contains supplementary material, which is available to authorized users.

## Background

While responsible conduct of research is expected of scientists [1–3], breaches of research integrity occur and some of these may be alarmingly common. The principles are made explicit in many codes of conduct [4–8]. These codes typically are “aspirational” in the sense that they focus on a description of the virtues and values scientists ought to live up to [9, 10]. Codes of conduct are often quite vague about the dos and don’ts and commonly do not try to make the norms of scientific research explicit in operational terms by listing the specific behaviors to adopt or to avoid.

In fact, there is a whole spectrum of minor and major research misbehaviors. At the extreme end of the spectrum, there are clear instances of research misconduct, like fabrication, falsification, and plagiarism (FFP). These “major” misbehaviors are clearly wrong and are typically committed intentionally. Most codes of conduct equal breaches of research integrity to committing research misconduct and try to distinguish this from “minor offences,” usually called questionable research practices (QRPs) or “sloppy science.” QRPs thus occupy an important part of the continuum with on one end research misconduct and on the other end responsible conduct of research. QRPs often concern trespasses of methodological principles that threaten the relevance, validity, trustworthiness, or efficiency of the study at issue [10] and may be committed intentionally or unintentionally. In the latter case, scientists do not know or do not agree that the behavior at issue is undesirable and should be avoided. In short, research misconduct as well as sloppy science threaten the validity of scientific knowledge and may also inflict damage to the trust between scientists and, if revealed in the media, may also damage public confidence in science.

The total harm caused by a specific research misbehavior depends on the frequency of its occurrence and the impact when it occurs. Furthermore, the impact is difficult to assess empirically and may vary between instances. This impact can consist of negative consequences for the validity of knowledge (“impact on truth”) and for the trust between scientists (“impact on trust”). Since the frequency of occurrence of most QRPs is probably much higher than the frequency of FFP, on the aggregate, QRPs may be much more detrimental than FFP [11, 12]. A number of surveys have focused on the self-reported prevalence of major and minor research misbehaviors [13–15]. A meta-analysis of 21 surveys concludes that 2% of the participants admit to have fabricated or falsified data at least once during the last 3 years [16]. The self-reported 3-year prevalence of questionable research practices varied widely across studies, with an unweighted mean of 34%. These surveys all focus on specific research misbehaviors, using partly overlapping but also different or differently formulated items.

Most researchers, when asked to give examples of breaches of research integrity will mention fabrication, falsification, and plagiarism, whereas only few can mention more than some QRP items. To date, there is no acknowledged and comprehensive list of major and minor misbehaviors available, which makes it rather laborious for educators and research leaders to acquire an adequate overview. Furthermore, the many forms of research misbehavior have to our knowledge not been ranked. Our first aim in this study is to present a comprehensive list of misbehaviors, acknowledged by experts. Our second study aim is to provide expert-based rankings of these specific research misbehaviors according to their views on and perceptions of the frequency of occurrence, preventability, impact on truth (validity), impact on trust between scientists, and combinations of these. We also will explore whether rankings differ between disciplinary fields.

## Methods

We drafted a list of major and minor research misbehaviors in five steps. Firstly, a comprehensive list of more than 100 items and sub-items was compiled from the literature, codes of conduct, guidelines, and our own experiences. This first list covered all the major and minor deviations from responsible conduct of research we could think of. Secondly, we shortened the list to 60 items by eliminating redundancies, merging items, rephrasing some of the items, and reformulating a number of items to make them relevant for all empirical research. Thirdly, this list was tried out by a group of 15 experienced researchers drafting a national research program on fostering responsible research practices in the Netherlands. In an interactive workshop, the items were ranked and comments on the formulation and the relevance of the items plus suggestions for items that also should be included were made. This resulted in some changes in the item list. Fourthly, we invited a selected group of 60 keynote speakers and session chairs 6 months before the 4th World Conference on Research Integrity (4th WCRI), Rio de Janeiro, 2015. In a web-based survey, they were asked to estimate frequency of occurrence and impact on truth and trust of all items, to suggest lacking items, and on the formulation of the items. Fifthly, the 34 respondents were invited (26 attended) for a workshop at the 4th WCRI which resulted in the list of items used in the project reported on in this publication. Also, the study design and the formulations of the survey questions and answer options were based on the workshop.

The authors interactively reached consensus on the allocation of the items to one of four topic domains: study design (items that concern the phase before the start of data collection), data collection (items that concern the phase of data collection), reporting (items that concern data-analysis and reporting the results of the study), and collaboration (items that concern obligations towards colleagues and science as a whole).

For our survey, we excluded the experts who had been involved in the formulation of the list of 60 items. Email addresses of the 1353 (1345 after omitting clearly incorrect email addresses) other participants of at least one of the four past World Conferences of Research Integrity [17] were randomly allocated to three groups [18]. Each participant received an email (see Additional file 1) with a link to a questionnaire (see Additional file 2) containing the 20 items of research misbehavior for that group. For each participant, we randomized the order of presentation of the items. We included only 20 misbehavior items in each questionnaire to avoid non-response due to an otherwise excessive length. Participants were asked about frequency, impact on truth, impact on trust, and preventability of each item on a 1–5 point scale (see Additional file 2). Impact on the aggregate level was operationalized by the product of frequency scores and truth, trust and preventability scores, respectively. Additionally, relevant demographic and professional background information was collected from all participants (see Additional file 2).

The invitational email (see Additional file 1) and subsequent reminders were sent in November and December 2015. The email included a link to the online questionnaire available on the website of SurveyMonkey [19] and another link to opt out. After 2 and 4 weeks, a reminder was sent to non-responders that had not opted out. Invitees that opted out were asked to disclose their reasons for declining participation and to provide basic background information. We emphasized that all questions pertained to the views of the respondents on the general situation in his or her disciplinary field and also that the survey concerns their personal subjective opinions on the items presented, that may have been formed by direct experience, stories from colleagues, and/or knowledge of the literature on research misbehavior (Additional file 3).

Data of the 60 items on frequency, impact on truth and trust, and preventability were exported from SurveyMonkey in SPSS format. Data were then read into STATA 13.1 using StatTransfer (version 12-64). Simple descriptive analyses were performed using STATA 13.1. The ranking was based on the mean scores on frequency, impact on truth, and impact on trust of the 60 items. The item means of the products of the scores for frequency and for impact on truth, of frequency and impact on trust, and of frequency and preventability were also calculated, and the items were then ranked accordingly using STATA 13.1.

## Results

Response was 17% of those who were sent the invitational email, 20% of the valid email addresses, 33% of the emails opened, and 77% of the links to the survey opened (see Table 1). Thirty-three invitees (5% of those who opened the invitational e-mail) disclosed their reasons for declining participation and provided basic background information, 39% of them argued that they were not involved in the area of research on research misbehaviors as the main reason for non-participation, while 24% reported no professional link to the area of research integrity.

**Table 1:** Response rates to the survey

	Number	Response rate
Invitational e-mails sent	1345	17% (227/1345)
Valid emails addresses	1131	20% (227/1131)
Emails opened	693	33% (227/693)
Links to survey opened	293	77% (227/293)
Participants that completed survey	227	

Thirty-five percent of the 227 respondents did not provide data on the characteristics listed in Table 2. Fifty-eight percent of the participants who did had a biomedical background and 19% came from the social sciences. On average, the participants of the survey worked in Academia for two decades or more, and the majority came from Western Europe and North America.

**Table 2:** Characteristics of survey participants

Male (%)	53
Age (years (sd))	53 (12.7)
Region (%)	
Western Europe	31
North America	28
South America	20
Asia	7
Eastern Europe	5
Africa	5
Australia/New Zealand/Pacific	4
Setting (%)	
University or university medical center	70
Non-profit research institute	8
For-profit research institute	2
Funding organization	2
Government	7
Academic publisher	5
Other	6
Job title (%)	
Full professor	25
Associate professor	12
Assistant professor	7
PhD student	7
Postdoc	1
Policy maker/policy advisor	14
Administrator	7
Other	27
Disciplinary field (%)	
Biomedical sciences	58
Social sciences	19
Natural sciences	15
Humanities	8
Holds PhD degree (%)	68
Years since PhD degree (sd)	23 (13.0)

Thirty-five percent of the 227 respondents did not answer these questions

*sd* standard deviation

Table 3 shows the top 5 of the 60 research misbehavior items when ranked according to perceived frequency of occurrence, assumed impact on truth and trust, the putative preventability, and the products of frequency and impact on truth, trust, and preventability, respectively. Additional file 4 shows the rankings for all 60 items, including the ranking of the summary priority scores. Additional file 5 shows all scores and the number of respondents per item, plus the views on how these research misbehaviors might be prevented.

**Table 3:** Top 5 rankings according to frequency, impact on truth and trust, and preventability

Rank number	Frequency (1–5)	Mean score (95% CI^a^)
1	Selectively cite to enhance your own findings or convictions (R)	3.53 (3.26–3.80)
2	Insufficiently supervise or mentor junior coworkers (C)	3.46 (3.18–3.74)
3	Not publish a valid “negative” study (R)	3.39 (3.09–3.70)
4	Demand or accept an authorship for which one does not qualify (C)	3.35 (3.02–3.69)
5	Selectively cite to please editors, reviewers, or colleagues (R)	3.29 (2.95–3.63)
Rank number	Impact on truth (1–5)	Mean score (95% CI)
1	Fabricate data (D)	4.63 (4.43–4.84)
2	Selectively delete data, modify data or add fabricated data after performing initial data-analyses (R)	4.36 (4.11–4.62)
3	Modify the results or conclusions of a study due to pressure of a sponsor (R)	4.35 (4.13–4.59)
4	Choose a clearly inadequate research design or using evidently unsuitable measurement instruments (S)	4.18 (3.93–4.42)
5	Conceal results that contradict your earlier findings or convictions (R)	4.04 (3.78–4.31)
Rank number	Impact on trust (1–5)	Mean score (95% CI)
1	Fabricate data (D)	4.70 (4.51–4.89)
2	Selectively delete data, modify data, or add fabricated data after performing initial data-analyses (R)	4.48 (4.28–4.69)
3	Modify the results or conclusions of a study due to pressure of a sponsor (R)	4.40 (4.15–4.66)
4	Review your own papers (C)	4.08 (3.63–4.52)
5	Unfairly review papers, grant applications or colleagues applying for promotion (C)	4.06 (3.79–4.34)
Rank number	Preventability (1–5)	Mean score (95% CI)
1	Ignore substantial safety risks of the study to participants, workers, or environment (S)	3.91 (3.58–4.25)
2	Review your own papers (C)	3.88 (3.47–4.30)
3	Ignore basic principles of quality assurance (D)	3.83 (3.61–4.05)
4	Use published ideas or phrases of others without referencing (C)	3.81 (3.55–4.08)
5	Inadequately handle or store data or (bio)materials (D)	3.79 (3.50–4.08)
Rank number	Product of frequency and impact on truth (1–25)	Mean score (95% CI)
1	Insufficiently supervise or mentor junior coworkers (C)	12.59 (11.29–13.89)
2	Insufficiently report study flaws and limitations (R)	12.32 (10.99–13.65)
3	Keep inadequate notes of the research process (D)	12.18 (10.57–13.78)
4	Turn a blind eye to putative breaches of research integrity by others (C)	12.12 (10.69–13.56)
5	Ignore basic principles of quality assurance (D)	12.04 (10.72–13.36)
Rank number	Product of frequency and impact on trust (1–25)	Mean score (95% CI)
1	Use published ideas or phrases of others without referencing (C)	12.08 (10.66–13.50)
2	Insufficiently report study flaws and limitations (R)	12.04 (10.68–13.41)
3	Turn a blind eye to putative breaches of research integrity by others (C)	11.96 (10.43–13.49)
4	Insufficiently supervise or mentor junior coworkers (C)	11.81 (10.55–13.07)
5	Ignore basic principles of quality assurance (D)	11.76 (10.40–13.11)
Rank number	Product of frequency and preventability (1–25)	Mean score (95% CI)
1	Insufficiently supervise or mentor junior coworkers (C)	12.96 (11.57–14.36)
2	Inadequately handle or store data or (bio)materials (D)	11.97 (10.22–13.72)
3	Use published ideas or phrases of others without referencing (C)	11.91 (10.49–13.32)
4	Keep inadequate notes of the research process (D)	11.88 (10.37–13.40)
5	Ignore basic principles of quality assurance (D)	11.40 (10.11–12.68)

95% *CI* 95% confidence interval, *R* item from the domain reporting, *C* item from the domain collaboration, *D* item from the domain data collection, *S* item from the domain study design

Selective citing features twice in the frequency top 5. Also insufficient supervision, not publishing a negative study and honorary authorship are believed to be highly prevalent. Our respondents agree that fabrication and falsification are the largest threats to validity if they occur. Furthermore, yielding to pressure by sponsors, using an inadequate study design and concealing unwelcome results are thought to compromise truth-finding. The same items—in a slightly different order—feature in the impact on trust top 5. The ranking of the five most important preventable items contains mainly trespasses of rules that could be maintained more strictly and that concern safety risks, handling and storage of data and (bio)materials, and quality assurance in general. Also, plagiarism and reviewing one’s own papers are considered to be preventable.

Insufficient supervision and mentoring rank highest in two of the aggregate rankings presented in Table 3, suggesting that this item is not only very important but also preventable. Also, keeping inadequate notes of the research process and ignoring basic principles of quality assurance are in both these top 5’s, which makes them additional candidates for intervention. The ranking based on the product of frequency and truth scores additionally emphasizes the importance of insufficiently reporting study flaws and limitations and of ignoring breaches of research integrity made by others. The ranking on the product of frequency and preventability scores also suggest that inadequate handling and storage of data and (bio)materials as well as plagiarism are important candidates for preventive action. An item concerning plagiarism is number one of the product ranking of frequency and trust and number three of the product ranking of frequency and preventability.

In Table 4, we summarize the data on the three forms of research misconduct: fabrication, falsification, and plagiarism. These items are ordered according to their increasing (perceived) frequency. While fabrication and falsification are believed to occur (very) infrequently, the plagiarism items are perceived to be more common. The impact on truth scores clearly shows that fabrication and falsification are—when they occur—believed to be the most severe validity threats by far. In summary, the ranking based on the product of frequency and impact on truth scores suggests that the “deadly sins” of research integrity contained in Table 4 rank only low to moderate and do not feature in the top 15.

**Table 4:** Ranking of research misconduct items: fabrication, falsification, and plagiarism

Item	Frequency Mean score (rank number)	Impact on truth Mean score (rank number)	Frequency × truth Mean score (rank number)
Fabricate data	1.88 (59)	4.63 (1)	8.82 (34)
Selectively delete data, modify data, or add fabricated data after performing initial data-analyses (F)	2.22 (50)	4.37 (2)	9.68 (24)
Delete data before performing data analysis without disclosure (F)	2.48 (45)	4.02 (6)	10.10 (19)
Duplicate publication without disclosure (P)	2.74 (36)	2.65 (49)	7.08 (48)
Re-use of previously published data without disclosure (P)	2.78 (29)	2.91 (46)	8.56 (36)
Use unpublished phrases or ideas of others without their permission (P)	2.92 (21)	2.96 (41)	8.84 (33)
Re-use parts of your own publications without referencing (P)	3.04 (15)	2.37 (55)	7.20 (46)
Use published phrases or ideas of others without referencing (P)	3.11 (12)	2.94 (43)	9.55 (26)

*F* falsification, *P* plagiarism

In a secondary analysis, we looked at the differences between disciplinary fields (see Additional file 6). For the humanities, we had not enough respondents for a meaningful comparison. It seems that in the biomedical sciences, selective citation is perceived to be more common than in the social sciences, and in the social sciences, reporting misbehaviors are believed to be more frequent. The differences between the rankings for biomedical and natural sciences appear to be small.

## Discussion

The non-ranked list of 60 research misbehaviors (see Additional file 3) has already been useful in our hands to show young as well as senior researchers that there are many possibilities to improve their research. Specifying the don’ts for educational purposes is in line with Mazar and Ariely’s recommendation to leave little room for rationalizing dishonesty [20].

We identified the research behaviors that were perceived to be major problems, by individuals who are likely to be experts and who may have had direct experience with issues relating to research integrity. It remains to be assessed how common these behaviors really are and whether the rankings reflect their actual gravity. We do not know what proportion of these conference participants are actually “experts” in judging the ranking of the behaviors about which our survey queried them. It is interesting to note that more than a quarter (27%) of our respondents reported their job title as “Other,” which might be due to an overrepresentation of academic job titles among the answer options. Therefore, we know little about the “credentials” of this group pertinent to the survey questions.

To our knowledge, this is the first attempt to rank a comprehensive list of major and minor research misbehaviors that covers all disciplinary fields. However, a Delphi survey among 40 experts identified types of scientific misconduct that would most likely bias the results and conclusions of a clinical trial [21]. That study, too, did not rank fabrication and falsification highly because the Delphi panel—like our respondents—judged their frequency of occurrence to be very low. A substantial number of the resulting list of 60 items was similar to ours. The list of 13 items that >50% of the Delphi panel thought to be likely or very likely to occur contains multiple forms of selective reporting and undisclosed data-driven statistical analyses.

The response (17%) was low, although some email addresses were invalid and only about half of the emails were opened. We were disappointed that only 42% (293/693) of those who opened the invitational email also opened the survey. These 400 invitees based their decision not to participate in the survey exclusively on the information contained in the invitational email (see Additional file 1). Some, unknown, proportion of these individuals may have decided that they lacked sufficient knowledge, experience or expertise to answer the survey questions adequately, in which case their decision not to participate would be quite appropriate, and not seen as a threat to the validity of the survey results.

Readers should bear in mind a number of other limitations inherent to our approach. We collected data on the personal views on the frequency and the impact of a series of major and minor research misbehaviors of a convenience sample consisting of participants of one or more World Conferences on Research Integrity. These views may be formed by direct experience, stories from colleagues, or knowledge of the literature on research misbehavior. We have no means to validate these views with more objective evidence on the occurrence and impact of the major and minor research misbehaviors we studied. Furthermore, although we tried to formulate the 60 items and our survey questions and answer options as unambiguously as possible, we do not know if the respondents, with their wide range of disciplinary backgrounds, had a common understanding of the issues raised and were not unduly influenced by the way we formulated the invitational email and the survey questions. Our numbers turned out to be too low to analyze heterogeneity of rankings for, e.g., disciplinary fields, geographical regions, and job titles. We cannot exclude that these characteristics influenced the rankings, for instance, when the many professors and the few PhD students in our sample would have different opinions on authorship. Another limitation is that our survey focused on trust between scientists and did not study the impact on trust in science in general. Finally, the interpretation of 95% confidence interval (CI) in a convenience sample with a low response rate is a bit problematic, although it is not unusual to present the 95% CI as an indicator of precision in situations other than inferences from a random sample to the source population.

A recent Cochrane review identified 31 studies on the effects on research behavior and researchers’ attitudes of educational and policy interventions [22] and made it clear that there is hardly any convincing evidence for an effect on research integrity or responsible conduct of research. Most studies were found to be of (very) low quality and many concerned training programs to avoid plagiarism. Taken together, the authors of the review conclude that the effects of educational and policy interventions are uncertain with a possible exception for training about plagiarism that uses practical exercises and text-matching software. Unfortunately, items concerning plagiarism scored low to very low on truth-based rankings in our survey.

The aggregate rankings in Table 3 place a great responsibility for prevention on supervisors and mentors. We doubt that supervisors and mentors in general are sufficiently aware of the wide spectrum of specific behaviors to be avoided our list stipulates (see Additional file 3). We speculate that supervisors and mentors could do a much better job if they used such a list systematically in training, supervision, and mentoring. We also believe that our list offers a clear and pragmatic view on the origin of a multitude of everyday research dilemmas because many of the don’ts can serve to unacceptable polishing of research findings. Thus our list can serve as a sobering “writing on the wall” for scientists who are under the spell of perverse incentives to prioritize high publication and citation rates for boosting their career, rather than devote themselves to the production of valid and trustworthy knowledge. Recently, Darwinian evolution theory was used to explain why sloppy science is a winning strategy to survive in modern science [23], and our findings also add to the current argumentation for changing the selective forces and to make the incentives of the science system less perverse by making the reward criteria more diverse [24]. Finally, we would like to emphasize that all attempts to fight sloppy science and worse should ideally be accompanied by sound evaluation to assess their effects [25, 26].

## Conclusions

Our ranking results seem to suggest that selective reporting, selective citing, and flaws in quality assurance and mentoring are the major evils of modern research. A picture emerges not of concern about wholesale fraud but of profound concerns that many scientists may be cutting corners and engage in sloppy science, possibly with a view to get more positive and more spectacular results that will be easier to publish in a high-impact journal and will attract many citations. In the fostering of responsible conduct of research, we recommend to develop interventions that actively discourage the high-ranking misbehaviors from our study.

## Additional files

Additional file 1: Invitation to participate. (PDF 53 kb)

Additional file 2: Survey questions. (PDF 76 kb)

Additional file 3: Major and minor misbehavior items. (PDF 81 kb)

Additional file 4: Rankings of major and minor misbehaviors. (PDF 73 kb)

Additional file 5: Results of the survey. (PDF 79 kb)

Additional file 6: Top 5 rankings for disciplinary fields. (PDF 111 kb)

## Abbreviations

FFP: Fabrication, falsification, and plagiarism
QRP: Questionable research practices
