# Correction to: ranking major and minor research misbehaviors: results from a survey among participants of four World Conferences on Research Integrity

**DOI:** 10.1186/s41073-019-0072-8

**Published:** 2019-06-28

**Authors:** Lex M. Bouter, Joeri Tijdink, Nils Axelsen, Brian C. Martinson, Gerben ter Riet

**Affiliations:** 1 Department of Epidemiology and Biostatistics, VU University Medical Center, Amsterdam, The Netherlands; 2 Department of Philosophy, Faculty of Humanities, Vrije Universiteit, Amsterdam, The Netherlands; 3 Department of Internal Medicine, VU University Medical Center, Amsterdam, The Netherlands; 4 Office of Research Integrity, Statens Serum Institut, Copenhagen, Denmark; 5 Department of Medicine, HealthPartners Institute and Minneapolis Veterans Affairs, Center for Chronic Disease Outcomes Research and University of Minnesota, Minneapolis, MN USA; 6 Department of General Practice, Academic Medical Center, University of Amsterdam, Amsterdam, The Netherlands

**Correction to: Research Integrity and Peer Review (2016) 1:17**

**https://doi.org/10.1186/s41073-016-0024-5**

Following publication of the original article [1], the authors reported that an incorrect version of Table 4.1 in Additional 4 has been published. A corrected version of Additional file 4 is attached to this Correction.

## Additional file

Additional file 4: Rankings of major and minor research misbehaviors. (PDF 78 kb)
