# Supplementary material for: Ranking major and minor research misbehaviors: results from a survey among participants of four World Conferences on Research Integrity
Source: Res Integr Peer Rev. 2016 Nov 21;1:17. doi: 10.1186/s41073-016-0024-5 (PMC5803629; doi:10.1186/s41073-016-0024-5)
Supplement: Supplementary file 1 — Invitation to participate. (PDF 53 kb) [file 41073_2016_24_MOESM1_ESM.pdf]

Additional file 1: Invitation to participate

Dear colleague,

We would like to invite you to take part in our web-based survey. We write to you because you participated in one or more of the past four World Conferences on Research Integrity. We are interested in your personal views on the frequency and the impact of a series of major and minor research misbehaviors. Your views may be formed by direct experience, stories from colleagues and/or knowledge of the literature on research misbehavior. We invite you to answer our questions for the disciplinary field you are most familiar with.

It is obvious that research misconduct can do a lot of damage. But perhaps 'sloppy science' due to questionable research practices does even more harm on the aggregate than intentional deceit. The total harm that is done by a specific form of research misbehavior depends on many factors, including its frequency and on the impact when it occurs.

We will look at two aspects of impact: the negative consequences (1) for the validity of the findings of the study at issue and (2) for the general level of trust between scientists, if the behavior were to become known. We will also ask you whether you believe that the specific misbehaviors can be prevented (e.g. by education, codes, guidelines, infrastructure, or cultural interventions), and which items deserve high priority in fostering responsible conduct of research.

A pilot version of the survey was send to 59 experts and the results were discussed in a workshop during the 4th World Conference on Research Integrity. This led to the current revised version of the questionnaire.

We want to collect views on 60 specific research misbehavior items. For the sake of your valuable time each invitee will be asked about a random subset of 20 of these items. This will take at most 20 minutes.

Here you'll find the link to the survey: < link to survey>.

We really hope you will accept our invitation. Your help is essential in ranking the research misbehavior items. We will inform participants about our findings. And we will explore the idea to organize a second workshop during the 5th World Conference on Research Integrity in Amsterdam, May 2017.

If you decide not to participate in our survey, please fill in this ultra-brief questionnaire for non-respondents (½ minute) < link to non-respondents-survey >.

Please respond to our invitation before <date>.

Best regards, also on behalf of Joeri Tijdink, Nils Axelsen, Brian Martinson, Daniele Fanelli and Gerben ter Riet ,

Lex Bouter

NB. Your anonymity is well protected. The research team has no possibility to link your responses to your e-mail addresses.
