# Supplementary material for: Ranking major and minor research misbehaviors: results from a survey among participants of four World Conferences on Research Integrity
Source: Res Integr Peer Rev. 2016 Nov 21;1:17. doi: 10.1186/s41073-016-0024-5 (PMC5803629; doi:10.1186/s41073-016-0024-5)
Supplement: Supplementary file 2 — Survey questions. (PDF 76 kb) [file 41073_2016_24_MOESM2_ESM.pdf]

Additional file 2: Survey questions

General questions

1. Which is the disciplinary field you are most familiar with?
    - a. Natural sciences (e.g. engineering, chemistry, mathematics)
    - b. Biomedical sciences (e.g. medicine, dentistry, veterinary medicine)
    - c. Social sciences (e.g. sociology, psychology, economics)
    - d. Humanities (e.g. history, linguistics, philosophy)

2. In what type of setting do you primarily work?
    - a. University or University Medical Center
    - b. Non-profit research institute
    - c. For-profit research institute or company
    - d. Funding organization
    - e. Government
    - f. Academic Publisher
    - g. Other (please specify):________________________

3. What is your job or job title?
    - a. PhD student
    - b. Postdoc
    - c. Assistant Professor
    - d. Associate Professor
    - e. Full Professor
    - f. Policymaker/policy advisor
    - g. Administrator
    - h. Other (please specify): _______________________

4. In which part of the world is your professional life predominantly based?
    - a. North America
    - b. South America
    - c. Western Europe
    - d. Eastern Europe
    - e. Africa
    - f. Asia
    - g. Australia/ New Zealand / Pacific

5. What is your gender?
    - a. Female
    - b. Male

6. What is your age?

    _ _ years

7. How many years have you been teaching and/or doing research (including PhD years)?

    _ _ years

8. When did you receive your PhD degree?
    - a. __ please specify in what year, e.g. 1990
    - b. __ not Applicable

9. On average, how many days a week do you spend on research and on teaching?
    - a. _ days on teaching

b. _ days on research

10. Are you professionally involved in one or more of the following activities?

a. _ teaching about research integrity and responsible conduct of research
b. _ doing research on research integrity and responsible conduct of research
c. _ handling allegations of breaches of research integrity
d. _ formulating and implementing policies about research integrity and responsible conduct of research
e. _ other, please specify

Instruction

For each item, please answer the following questions for the general situation in the disciplinary field (biomedical sciences, social sciences, natural sciences, or humanities) you're most familiar with. We are interested in your personal views and opinions. Clearly these may be based on direct experience, stories from colleagues and/or knowledge of the literature on research misbehavior.

Questions for each item

1. In my disciplinary field this form of research misbehavior does NOT apply

2. How often does this form of research misbehavior occur in the disciplinary field you're most familiar with?

very rarely (1) - rarely (2) - regularly (3) – often (4) – very often (5)

3. If it occurs, how large will its impact be on the validity of the findings of the study at issue?

very small (1) - small (2) – medium (3) – large (4) – very large (5)

4. If it occurs and becomes known, how large will its impact be on the general level of trust between scientists?

very small (1) - small (2) – medium (3) – large (4) – very large (5)

5. To what extent can this research misbehavior be prevented?

very small (1) - small (2) – medium (3) – large (4) – very large (5)

6. How can this item be prevented best? (choose only one)

_ education
_ code or guideline
_ infrastructure
_ audits
_ properly aligned incentives
_ intervention at the level of the research culture
_ other intervention (please specify): _______________

7. In summary, how do you rate the priority of this item for fostering responsible conduct of research?
very low (1) – low (2) – medium (3) – high (4) – very high (5)
