# Supplementary material for: Ranking major and minor research misbehaviors: results from a survey among participants of four World Conferences on Research Integrity
Source: Res Integr Peer Rev. 2016 Nov 21;1:17. doi: 10.1186/s41073-016-0024-5 (PMC5803629; doi:10.1186/s41073-016-0024-5)
Supplement: Supplementary file 3 — Major and minor misbehavior items. (PDF 81 kb) [file 41073_2016_24_MOESM3_ESM.pdf]

Additional file 3: Major and minor misbehavior items

Study design
1. Propose study questions which are clearly irrelevant<br>[including questions that have already been or could be answered adequately by a systematic review of the literature]<br><br>2. Choose a clearly inadequate research design or using evidently unsuitable measurement instruments<br>[which will not lead to a valid, reproducible and efficient answer to the main study question, taking into account the state-of-the-art in the field at issue]<br><br>3. Present grossly misleading information in a grant application<br><br>4. Write no or a clearly inadequate research protocol<br>[in which essential details are lacking]<br><br>5. Ignore substantial safety risks of the study to participants, workers or environment<br><br>6. Ignore substantial risks of the expected findings for society or environment<br><br>7. Importantly change the research design during the study without disclosure<br>[or – if applicable- without permission of sponsor, Institutional Review Board or Institutional Animal Care and Use Committee]<br><br>8. Give insufficient attention to the equipment, skills or expertise which are essential to perform the study
Data collection
9. Collect more data after noticing that the results are almost statistically significant<br>[unless specified in a predefined adequate plan for interim analysis – also called 'peeking']<br><br>10. Fabricate data<br><br>11. Stop data collection earlier than planned because the results are already statistically significant<br>[unless predefined stopping rules are implemented appropriately - also called 'peeking']<br><br>12. Not adhere to pertinent laws and regulations<br>[including the laws and regulations for human and animal studies, safety regulations, good clinical practice, good laboratory practice etc.]<br><br>13. Inadequately handle or store data or (bio)materials<br>[including archiving for an appropriate period]<br><br>14. Keep inadequate notes of the research process<br>[with (digital) lab journals or its equivalent in other types of research]<br><br>15. Ignore basic principles of quality assurance
Reporting

16. Report on data-driven hypotheses without disclosure
['HARKing' - Hypothesizing After Results are Known - typically with a view to make results to appear more spectacular ('Chrysalis effect')]

17. Delete data before performing data analysis without disclosure

18. Selectively delete data, modify data or add fabricated data after performing initial data-analyses
[in other words: falsification or fabrication of data]

19. Perform data-analyses not stated in the study protocol without disclosure
[or in predefined data-analysis plan – also called 'Significance chasing', 'P-hacking', 'data dredging', 'fishing expedition' or explorative subgroup analyses]

20. Report an incorrect downwardly rounded p-value
[e.g. by reporting a p value of .054 as being less than .05]

21. Not report all study protocol-stipulated results
[in the aggregate of all published reports on the study at issue]

22. Not publish a valid 'negative' study
[in a form that is publicly available or accessible behind a paywall (article, report, website etc.)]

23. Report an unexpected finding as having been hypothesized from the start

24. Conceal results that contradict your earlier findings or convictions

25. Not report clearly relevant details of study methods

26. Not report replication problems

27. Selectively cite to enhance your own findings or convictions

28. Selectively cite to please editors, reviewers or colleagues

29. Selectively cite or cite your own work to improve citation metrics
[e.g. Impact Factor, H-index]

30. Let your convictions influence the conclusions substantially

31. Insufficiently report study flaws and limitations

32. Spread study results over more papers than needed
['salami slicing']

33. Duplicate publication without disclosure

34. Re-use of previously published data without disclosure
[which may lead to double counting in meta-analyses]

35. Modify the results or conclusions of a study due to pressure of a sponsor
[commercial or not-for-profit funder of the study]

36. Failure to disclose a sponsor of the study

37. Failure to disclose a relevant financial or intellectual conflict of interest

[in publications, when reviewing grant proposals, or evaluating persons or institutions]

38. Handle existing conflicts of interest inadequately

39. Communicate results to the general public before a peer reviewed publication is available

40. Deliberately communicate findings inaccurately in the media or during presentations

41. Make no clear distinction between personal views and professional comments

Collaboration

42. Take no full responsibility for the integrity of the research project and its reports

43. Refuse to share data with bona fide colleagues

44. Turn a blind eye to putative breaches of research integrity by others

45. Refuse to respond to an allegation of a breach of research integrity

46. Use unpublished ideas or phrases of others without their permission
[e.g. from reviewing manuscripts or grant applications, or from conference presentations - this is one of the forms plagiarism can take]

47. Use published ideas or phrases of others without referencing
[this is one of the forms plagiarism can take]

48. Re-use parts of your own publications without referencing
['self-plagiarism']

49. Unfairly review papers, grant applications or colleagues applying for promotion

50. Review your own papers

51. Demand, accept or offer substantial gifts for doing a favor
[e.g. authorship, promotion, access to data, favorable review or recommendation]

52. Insufficiently supervise or mentor junior coworkers

53. Be grossly unfair to your collaborators
[e.g. in terms of a just balance of benefits and burdens, including giving those who deserve the opportunity to qualify as author]

54. Add an author who doesn't qualify for authorship
['honorary or gift authorship']

55. Demand or accept an authorship for which you don't qualify
['honorary or gift authorship']

56. Omit a contributor who deserves authorship
['ghost authorship']

57. Not acknowledge contributors who do not qualify for authorship

58. Not ask permission from contributors for the wording of the acknowledgement

59. Not share reviewers’ comments with all co-authors

60. Submit or resubmit a paper or grant application without consent from all authors
