# Supplementary material for: Ranking major and minor research misbehaviors: results from a survey among participants of four World Conferences on Research Integrity
Source: Res Integr Peer Rev. 2016 Nov 21;1:17. doi: 10.1186/s41073-016-0024-5 (PMC5803629; doi:10.1186/s41073-016-0024-5)
Supplement: Supplementary file 4 — Rankings of major and minor misbehaviors. (PDF 73 kb) [file 41073_2016_24_MOESM4_ESM.pdf]

Additional file 4: Rankings of major and minor research misbehaviors

Additional file 4.1: Ranking according to frequency

Rank	Research Misbehavior	Frequency (mean)	Lower 95% confidence limit	Upper 95% confidence limit
1	Selectively cite or cite your own work to improve citation metrics (R)	3.70	3.24	4.15
2	Demand or accept an authorship for which one does not qualify (C)	3.68	3.29	4.07
3	Selectively cite to enhance your own findings or convictions (R)	3.54	3.14	3.93
4	Add an author who doesn’t qualify for authorship (C)	3.54	3.09	3.98
5	Turn a blind eye to putative breaches of research integrity by others ..	3.50	3.00	4.00
6	Not publish a valid 'negative' study (R)	3.44	3.10	3.78
7	Insufficiently supervise or mentor junior coworkers (C)	3.41	3.01	3.82
8	Keep inadequate notes of the research process (D)	3.41	2.88	3.94
9	Inadequately handle or store data or (bio)materials (D)	3.36	2.86	3.86
10	Make no clear distinction between personal views and professional comm..	3.24	2.66	3.81
11	Insufficiently report study flaws and limitations (R)	3.21	2.89	3.54
12	Selectively cite to please editors, reviewers or colleagues (C)	3.21	2.75	3.66
12	Use published ideas or phrases of others without referencing (R)	3.21	2.82	3.60
14	Spread study results over more papers than needed (R)	3.17	2.78	3.55
15	Not report replication problems (R)	3.14	2.75	3.53
16	Ignore basic principles of quality assurance (D)	3.13	2.68	3.58
17	Perform data-analyses not stated in the study protocol without disclos..	3.13	2.71	3.54
18	Use unpublished ideas or phrases of others without their permission (C)	3.11	2.74	3.48
19	Collect more data after noticing that the results are almost statistic..	3.07	2.74	3.40
20	Report an unexpected finding as having been hypothesized from the star..	3.05	2.46	3.63
21	Not ask permission by contributors for the wording of the acknowledgem..	3.04	2.65	3.42
21	Let your convictions influence the conclusions substantially (R)	3.04	2.64	3.44
23	Not report clearly relevant details of study methods (R)	3.04	2.61	3.46
24	Report on data-driven hypotheses without disclosure (R)	3.00	2.57	3.43
24	Not report all study protocol-stipulated results (R)	3.00	2.61	3.39
26	Take no full responsibility for the integrity of the research project ..	2.95	2.51	3.39
27	Re-use parts of your own publications without referencing (R)	2.92	2.53	3.31
27	Omit a contributor who deserves authorship (C)	2.92	2.49	3.34
29	Refuse sharing data with bona fide colleagues (C)	2.88	2.46	3.31
30	Handle existing conflicts of interest inadequately (C)	2.87	2.46	3.28
31	Write no or a clearly inadequate research protocol (S)	2.84	2.44	3.24

32	Be grossly unfair to your collaborators (C)	2.79	2.37	3.22
32	Submit or resubmit a paper or grant application without consent from a..	2.79	2.46	3.12
34	Not acknowledge contributors who do not qualify for authorship (C)	2.74	2.35	3.13
35	Importantly change the research design during the study without disclo..	2.73	2.38	3.08
36	Unfairly review papers, grant applications or colleagues applying for ..	2.71	2.25	3.17
37	Failure to disclose a relevant financial or intellectual conflict of i..	2.69	2.31	3.07
38	Deliberately communicate findings inaccurately in the media or during ..	2.65	2.28	3.03
38	Re-use of previously published data without disclosure (R)	2.65	2.25	3.06
40	Duplicate publication without disclosure (R)	2.60	2.26	2.94
41	Conceal results that contradict your earlier findings or convictions (R)	2.59	2.26	2.93
42	Give insufficient attention to the equipment, skills or expertise whic..	2.59	2.19	2.99
43	Propose study questions which are clearly irrelevant (S)	2.55	2.07	3.03
44	Delete data before performing data analysis without disclosure (R)	2.45	2.01	2.90
45	Not share reviewers’ comments with all co-authors (C)	2.45	2.04	2.86
46	Stop data collection earlier than planned because the results are alre..	2.43	2.14	2.72
47	Choose a clearly inadequate research design or using evidently unsuita..	2.41	2.01	2.81
48	Selectively delete data, modify data or add fabricated data after perf..	2.33	1.97	2.70
49	Report an incorrect downwardly rounded p-value (R)	2.25	1.76	2.74
50	Failure to disclose a sponsor of the study (R)	2.24	1.94	2.54
51	Modify the results or conclusions of a study due to pressure of a spon..	2.23	1.82	2.63
52	Ignore substantial risks of the expected findings for society or envir..	2.22	1.79	2.66
53	Demand, accept or offer substantial gifts for doing a favor (C)	2.20	1.76	2.64
54	Communicate results to the general public before a peer reviewed publi..	2.19	1.79	2.59
55	Present grossly misleading information in a grant application (C)	2.13	1.82	2.44
56	Not adhere to pertinent laws and regulations (D)	2.13	1.78	2.47
57	Ignore substantial safety risks of the study to participants, workers ..	2.06	1.57	2.55
58	Refuse to respond to an allegation of a breach of research integrity (C)	1.95	1.60	2.31
59	Fabricate data (D)	1.93	1.56	2.29
60	Review your own papers (C)	1.83	1.43	2.24

Additional file 4.2: Ranking according to impact on validity

Rank	Research Misbehavior	Truth (mean)	Lower 95% confidence limit	Upper 95% confidence limit
1	Fabricate data (D)	4.63	4.43	4.84
2	Selectively delete data, modify data or add fabricated data after perf..	4.37	4.10	4.62
3	Modify the results or conclusions of a study due to pressure of a spon..	4.36	4.13	4.59
4	Choose a clearly inadequate research design or using evidently unsuita..	4.18	3.93	4.42
5	Conceal results that contradict your earlier findings or convictions (R)	4.04	3.78	4.31
6	Delete data before performing data analysis without disclosure (R)	4.02	3.71	4.34
7	Review your own papers (C)	4.00	3.57	4.43

7	Ignore basic principles of quality assurance (D)	4.00	3.79	4.21
9	Refuse to respond to an allegation of a breach of research integrity (C)	3.84	3.52	4.17
10	Keep inadequate notes of the research process (D)	3.82	3.56	4.09
11	Turn a blind eye to putative breaches of research integrity by others ..	3.78	3.52	4.04
12	Insufficiently report study flaws and limitations (R)	3.77	3.53	4.01
13	Let your convictions influence the conclusions substantially (R)	3.74	3.47	4.01
14	Not report replication problems (R)	3.73	3.39	4.06
15	Give insufficient attention to the equipment, skills or expertise whic..	3.70	3.47	3.93
16	Write no or a clearly inadequate research protocol (S)	3.66	3.33	3.99
17	Insufficiently supervise or mentor junior coworkers (C)	3.63	3.39	3.86
18	Not report all study protocol-stipulated results (R)	3.61	3.30	3.92
18	Not report clearly relevant details of study methods (R)	3.61	3.32	3.90
20	Unfairly review papers, grant applications or colleagues applying for ..	3.57	3.28	3.87
21	Handle existing conflicts of interest inadequately (C)	3.55	3.26	3.83
22	Failure to disclose a relevant financial or intellectual conflict of i..	3.53	3.24	3.82
23	Not adhere to pertinent laws and regulations (D)	3.51	3.17	3.86
24	Report an incorrect downwardly rounded p-value (R)	3.50	3.17	3.84
25	Deliberately communicate findings inaccurately in the media or during ..	3.47	3.10	3.85
26	Importantly change the research design during the study without disclo..	3.44	3.13	3.76
27	Perform data-analyses not stated in the study protocol without disclos..	3.37	2.99	3.76
28	Report on data-driven hypotheses without disclosure (R)	3.34	2.95	3.74
29	Present grossly misleading information in a grant application (C)	3.34	2.99	3.67
30	Selectively cite to enhance your own findings or convictions (R)	3.30	3.02	3.57
31	Inadequately handle or store data or (bio)materials (D)	3.29	2.91	3.67
32	Not publish a valid 'negative' study (R)	3.27	2.82	3.72
33	Ignore substantial risks of the expected findings for society or envir..	3.25	2.76	3.74
34	Stop data collection earlier than planned because the results are alre..	3.16	2.79	3.53
35	Ignore substantial safety risks of the study to participants, workers ..	3.15	2.69	3.60
36	Make no clear distinction between personal views and professional comm..	3.14	2.84	3.45
37	Collect more data after noticing that the results are almost statistic..	3.13	2.81	3.45
38	Failure to disclose a sponsor of the study (R)	3.09	2.75	3.43
39	Take no full responsibility for the integrity of the research project ..	3.00	2.68	3.32
40	Propose study questions which are clearly irrelevant (S)	2.97	2.61	3.33
41	Use unpublished ideas or phrases of others without their permission (C)	2.96	2.65	3.27
42	Refuse sharing data with bona fide colleagues (C)	2.96	2.62	3.29
43	Use published ideas or phrases of others without referencing (R)	2.94	2.63	3.26
44	Not share reviewers’ comments with all co-authors (C)	2.93	2.63	3.23
45	Demand, accept or offer substantial gifts for doing a favor (C)	2.92	2.54	3.29
46	Re-use of previously published data without disclosure (R)	2.91	2.58	3.25
47	Report an unexpected finding as having been hypothesized from the star..	2.83	2.40	3.25
48	Be grossly unfair to your collaborators (C)	2.74	2.44	3.04
49	Duplicate publication without disclosure (R)	2.65	2.28	3.02
50	Selectively cite or cite your own work to improve citation metrics (R)	2.63	2.39	2.88
51	Selectively cite to please editors, reviewers or colleagues (C)	2.59	2.28	3.00
52	Submit or resubmit a paper or grant application without consent from a..	2.47	2.14	2.80
53	Communicate results to the general public before a peer reviewed publi..	2.46	2.10	2.82
54	Spread study results over more papers than needed (R)	2.38	2.11	2.64
55	Re-use parts of your own publications without referencing (R)	2.37	2.10	2.64
56	Omit a contributor who deserves authorship (C)	2.30	2.05	2.55
57	Demand or accept an authorship for which one does not qualify (C)	2.29	1.98	2.61
58	Add an author who doesn’t qualify for authorship (C)	2.07	1.77	2.37
59	Not acknowledge contributors who do not qualify for authorship (C)	2.00	1.68	2.32
60	Not ask permission by contributors for the wording of the acknowledgem..	1.88	1.61	2.14

Additional file 4.3: Ranking according to impact on trust

Rank	Research Misbehavior	Trust (mean)	Lower 95% confidence limit	Upper 95% confidence limit
1	Fabricate data (D)	4.70	4.51	4.89
2	Selectively delete data, modify data or add fabricated data after perf..	4.48	4.28	4.69
3	Modify the results or conclusions of a study due to pressure of a spon..	4.40	4.15	4.66
4	Review your own papers (C)	4.08	3.63	4.52
5	Unfairly review papers, grant applications or colleagues applying for ..	4.06	3.79	4.34
6	Refuse to respond to an allegation of a breach of research integrity (C)	4.00	3.71	4.29
7	Delete data before performing data analysis without disclosure (R)	3.95	3.65	4.26
8	Ignore basic principles of quality assurance (D)	3.94	3.69	4.18
9	Conceal results that contradict your earlier findings or convictions (R)	3.94	3.67	4.20
10	Failure to disclose a relevant financial or intellectual conflict of i..	3.92	3.65	4.19
11	Ignore substantial risks of the expected findings for society or envir..	3.92	3.53	4.30
12	Turn a blind eye to putative breaches of research integrity by others ..	3.90	3.60	4.20

13	Use unpublished ideas or phrases of others without their permission (C)	3.90	3.63	4.16
14	Use published ideas or phrases of others without referencing (R)	3.81	3.54	4.10
15	Not adhere to pertinent laws and regulations (D)	3.80	3.48	4.12
16	Demand, accept or offer substantial gifts for doing a favor (C)	3.78	3.43	4.13
17	Deliberately communicate findings inaccurately in the media or during ..	3.75	3.42	4.08
18	Ignore substantial safety risks of the study to participants, workers ..	3.74	3.37	4.10
19	Present grossly misleading information in a grant application (C)	3.70	3.38	4.01
20	Insufficiently report study flaws and limitations (R)	3.68	3.41	3.95
21	Handle existing conflicts of interest inadequately (C)	3.68	3.39	3.97
22	Be grossly unfair to your collaborators (C)	3.63	3.33	3.92
23	Not report replication problems (R)	3.59	3.23	3.95
24	Report an incorrect downwardly rounded p-value (R)	3.59	3.23	3.95
25	Not report clearly relevant details of study methods (R)	3.59	3.32	3.85
26	Inadequately handle or store data or (bio)materials (D)	3.58	3.29	3.87
27	Not report all study protocol-stipulated results (R)	3.57	3.25	3.90
28	Refuse sharing data with bona fide colleagues (C)	3.55	3.23	3.87
29	Write no or a clearly inadequate research protocol (S)	3.50	3.21	3.79
30	Keep inadequate notes of the research process (D)	3.47	3.14	3.79
31	Let your convictions influence the conclusions substantially (R)	3.44	3.15	3.73
32	Give insufficient attention to the equipment, skills or expertise whic..	3.44	3.14	3.73
33	Choose a clearly inadequate research design or using evidently unsuita..	3.43	3.12	3.74
34	Insufficiently supervise or mentor junior coworkers (C)	3.40	3.17	3.64
35	Importantly change the research design during the study without disclo..	3.38	3.02	3.74
36	Not share reviewers’ comments with all co-authors (C)	3.34	3.02	3.67
37	Re-use of previously published data without disclosure (R)	3.31	2.99	3.63
38	Take no full responsibility for the integrity of the research project ..	3.30	3.01	3.60
39	Omit a contributor who deserves authorship (C)	3.30	2.98	3.61
40	Failure to disclose a sponsor of the study (R)	3.27	2.91	3.64
41	Report on data-driven hypotheses without disclosure (R)	3.26	2.95	3.56
42	Demand or accept an authorship for which one does not qualify (C)	3.20	2.92	3.47
42	Duplicate publication without disclosure (R)	3.20	2.88	3.51
44	Submit or resubmit a paper or grant application without consent from a..	3.19	2.88	3.50
45	Not publish a valid 'negative' study (R)	3.16	2.76	3.56
46	Selectively cite to enhance your own findings or convictions (R)	3.15	2.86	3.44
47	Make no clear distinction between personal views and professional comm..	3.09	2.83	3.36
48	Perform data-analyses not stated in the study protocol without disclos..	3.09	2.75	3.42
49	Add an author who doesn’t qualify for authorship (C)	2.98	2.67	3.30
50	Selectively cite or cite your own work to improve citation metrics (R)	2.98	2.72	3.24
51	Propose study questions which are clearly irrelevant (S)	2.97	2.69	3.25
52	Report an unexpected finding as having been hypothesized from the star..	2.94	2.56	3.33
53	Collect more data after noticing that the results are almost statistic..	2.94	2.61	3.26
54	Stop data collection earlier than planned because the results are alre..	2.86	2.53	3.20
55	Selectively cite to please editors, reviewers or colleagues (C)	2.86	2.54	3.19
56	Re-use parts of your own publications without referencing (R)	2.83	2.51	3.14
57	Not acknowledge contributors who do not qualify for authorship (C)	2.81	2.46	3.16
58	Communicate results to the general public before a peer reviewed publi..	2.76	2.35	3.16
59	Spread study results over more papers than needed (R)	2.63	2.35	2.90
60	Not ask permission by contributors for the wording of the acknowledgem..	2.59	2.27	2.90

Additional file 4.4: Ranking according to preventability

Rank	Research Misbehavior	Prevent-ability (mean)	Lower 95% confidence limit	Upper 95% confidence limit
1	Ignore substantial safety risks of the study to participants, workers ..	3.91	3.58	4.25
2	Review your own papers (C)	3.88	3.47	4.30
3	Ignore basic principles of quality assurance (D)	3.83	3.61	4.05
4	Use published ideas or phrases of others without referencing (R)	3.81	3.55	4.08
5	Inadequately handle or store data or (bio)materials (D)	3.79	3.50	4.08
6	Duplicate publication without disclosure (R)	3.79	3.53	4.04
7	Give insufficient attention to the equipment, skills or expertise whic..	3.77	3.54	4.00
8	Insufficiently supervise or mentor junior coworkers (C)	3.75	3.49	4.01
9	Submit or resubmit a paper or grant application without consent from a..	3.73	3.45	4.01
10	Choose a clearly inadequate research design or using evidently unsuita..	3.71	3.43	3.99
11	Write no or a clearly inadequate research protocol (S)	3.71	3.40	4.01
12	Keep inadequate notes of the research process (D)	3.70	3.47	3.93
13	Not report clearly relevant details of study methods (R)	3.67	3.42	3.93
14	Not share reviewers’ comments with all co-authors (C)	3.63	3.37	3.90
15	Report an incorrect downwardly rounded p-value (R)	3.62	3.27	3.98

16	Failure to disclose a sponsor of the study (R)	3.61	3.38	3.84
17	Ignore substantial risks of the expected findings for society or envir..	3.57	3.23	3.92
18	Re-use parts of your own publications without referencing (R)	3.55	3.31	3.79
19	Modify the results or conclusions of a study due to pressure of a spon..	3.54	3.25	3.83
20	Refuse to respond to an allegation of a breach of research integrity (C)	3.52	3.21	3.83
21	Not adhere to pertinent laws and regulations (D)	3.49	3.23	3.74
22	Importantly change the research design during the study without disclo..	3.49	3.17	3.80
23	Handle existing conflicts of interest inadequately (C)	3.45	3.19	3.71
24	Not report replication problems (R)	3.41	3.15	3.67
25	Failure to disclose a relevant financial or intellectual conflict of i..	3.41	3.17	3.64
26	Insufficiently report study flaws and limitations (R)	3.40	3.17	3.64
27	Not ask permission by contributors for the wording of the acknowledgem..	3.39	3.12	3.66
28	Re-use of previously published data without disclosure (R)	3.38	3.13	3.62
29	Be grossly unfair to your collaborators (C)	3.37	3.14	3.61
30	Not report all study protocol-stipulated results (R)	3.37	3.09	3.64
31	Present grossly misleading information in a grant application (C)	3.34	3.10	3.58
32	Fabricate data (D)	3.34	3.05	3.63
33	Omit a contributor who deserves authorship (C)	3.33	3.09	3.56
34	Delete data before performing data analysis without disclosure (R)	3.29	3.03	3.56
35	Spread study results over more papers than needed (R)	3.29	3.05	3.52
36	Not acknowledge contributors who do not qualify for authorship (C)	3.27	2.93	3.61
37	Turn a blind eye to putative breaches of research integrity by others ..	3.26	3.01	3.51
38	Take no full responsibility for the integrity of the research project ..	3.24	2.94	3.54
39	Not publish a valid 'negative' study (R)	3.24	2.89	3.59
40	Demand or accept an authorship for which one does not qualify (C)	3.24	2.93	3.54
41	Selectively delete data, modify data or add fabricated data after perf..	3.22	2.91	3.51
42	Add an author who doesn’t qualify for authorship (C)	3.21	2.88	3.54
43	Communicate results to the general public before a peer reviewed publi..	3.19	2.87	3.51
44	Demand, accept or offer substantial gifts for doing a favor (C)	3.19	2.89	3.49
45	Stop data collection earlier than planned because the results are alre..	3.17	2.82	3.51
45	Selectively cite to enhance your own findings or convictions (R)	3.17	2.90	3.43
47	Perform data-analyses not stated in the study protocol without disclos..	3.14	2.75	3.54
47	Use unpublished ideas or phrases of others without their permission (C)	3.14	2.82	3.46
47	Report on data-driven hypotheses without disclosure (R)	3.14	2.85	3.43
50	Selectively cite or cite your own work to improve citation metrics (R)	3.13	2.88	3.39
51	Deliberately communicate findings inaccurately in the media or during ..	3.13	2.79	3.46
52	Selectively cite to please editors, reviewers or colleagues (C)	3.11	2.78	3.45
53	Conceal results that contradict your earlier findings or convictions (R)	3.11	2.82	3.40
54	Refuse sharing data with bona fide colleagues (C)	3.07	2.71	3.42
55	Report an unexpected finding as having been hypothesized from the star..	3.06	2.66	3.45
56	Propose study questions which are clearly irrelevant (S)	3.06	2.69	3.42
57	Unfairly review papers, grant applications or colleagues applying for ..	3.02	2.72	3.32
58	Make no clear distinction between personal views and professional comm..	3.00	2.75	3.25
59	Let your convictions influence the conclusions substantially (R)	2.94	2.66	3.22
60	Collect more data after noticing that the results are almost statistic..	2.83	2.52	3.14

Additional file 4.5: Ranking according to product of frequency and impact on validity

Rank	Research Misbehavior	Product Truth (mean)	Lower 95% confidence limit	Upper 95% confidence limit
1	Insufficiently supervise or mentor junior coworkers (C)	12.59	11.29	13.89
2	Insufficiently report study flaws and limitations (R)	12.32	10.99	13.65
3	Keep inadequate notes of the research process (D)	12.18	10.57	13.78
4	Turn a blind eye to putative breaches of research integrity by others ..	12.13	10.69	13.56
5	Ignore basic principles of quality assurance (D)	12.04	10.72	13.36
6	Selectively cite to enhance your own findings or convictions (R)	11.70	10.38	13.01
7	Not publish a valid 'negative' study (R)	11.49	9.66	13.31
8	Let your convictions influence the conclusions substantially (R)	11.13	9.72	12.53

9	Not report clearly relevant details of study methods (R)	11.09	9.62	12.55
10	Inadequately handle or store data or (bio)materials (D)	10.87	9.12	12.62
11	Perform data-analyses not stated in the study protocol without disclos..	10.74	9.07	12.42
12	Not report replication problems (R)	10.51	9.05	11.98
13	Conceal results that contradict your earlier findings or convictions (R)	10.51	9.25	11.77
14	Choose a clearly inadequate research design or using evidently unsuita..	10.39	9.06	11.71
15	Handle existing conflicts of interest inadequately (C)	10.36	9.08	11.64
16	Not report all study protocol-stipulated results (R)	10.31	8.90	11.73
17	Write no or a clearly inadequate research protocol (S)	10.27	8.74	11.80
18	Unfairly review papers, grant applications or colleagues applying for ..	10.23	8.78	11.69
19	Delete data before performing data analysis without disclosure (R)	10.10	8.58	11.61
20	Report on data-driven hypotheses without disclosure (R)	9.97	8.41	11.53
21	Failure to disclose a relevant financial or intellectual conflict of i..	9.94	8.60	11.28
22	Make no clear distinction between personal views and professional comm..	9.81	8.44	11.18
23	Give insufficient attention to the equipment, skills or expertise whic..	9.80	8.35	11.25
24	Selectively delete data, modify data or add fabricated data after perf..	9.68	8.32	11.04
25	Importantly change the research design during the study without disclo..	9.56	8.28	10.83
26	Use published ideas or phrases of others without referencing (R)	9.55	8.23	10.87
27	Deliberately communicate findings inaccurately in the media or during ..	9.45	7.90	11.00
28	Propose study questions which are clearly irrelevant (S)	9.33	7.78	10.89
29	Modify the results or conclusions of a study due to pressure of a spon..	9.12	7.65	10.59
30	Collect more data after noticing that the results are almost statistic..	8.98	7.66	10.29
31	Selectively cite to please editors, reviewers or colleagues (C)	8.98	7.64	10.32
32	Selectively cite or cite your own work to improve citation metrics (R)	8.92	7.83	10.02
33	Use unpublished ideas or phrases of others without their permission (C)	8.84	7.59	10.08
34	Fabricate data (D)	8.82	7.69	9.94
35	Take no full responsibility for the integrity of the research project ..	8.69	7.41	9.97
36	Re-use of previously published data without disclosure (R)	8.56	7.24	9.87
37	Not adhere to pertinent laws and regulations (D)	8.53	7.15	9.92
38	Refuse sharing data with bona fide colleagues (C)	8.45	7.13	9.77
39	Refuse to respond to an allegation of a breach of research integrity (C)	8.40	7.06	9.74
40	Report an unexpected finding as having been hypothesized from the star..	8.35	6.59	10.12
41	Be grossly unfair to your collaborators (C)	8.30	7.09	9.51
42	Demand or accept an authorship for which one does not qualify (C)	8.27	6.96	9.59
43	Report an incorrect downwardly rounded p-value (R)	8.06	6.49	9.63
44	Spread study results over more papers than needed (R)	7.69	6.62	8.76
45	Stop data collection earlier than planned because the results are alre..	7.33	6.12	8.54
46	Re-use parts of your own publications without referencing (R)	7.20	6.10	8.30

47	Ignore substantial risks of the expected findings for society or envir..	7.11	5.66	8.57
48	Duplicate publication without disclosure (R)	7.08	5.83	8.33
49	Present grossly misleading information in a grant application (C)	6.88	5.86	7.90
50	Add an author who doesn't qualify for authorship (C)	6.73	5.59	7.86
51	Review your own papers (C)	6.64	5.33	7.95
52	Omit a contributor who deserves authorship (C)	6.40	5.46	7.35
53	Submit or resubmit a paper or grant application without consent from a..	6.36	5.34	7.38
54	Ignore substantial safety risks of the study to participants, workers ..	6.30	4.99	7.61
55	Not share reviewers' comments with all co-authors (C)	6.07	5.02	7.12
56	Failure to disclose a sponsor of the study (R)	5.98	4.92	7.04
57	Not acknowledge contributors who do not qualify for authorship (C)	5.77	4.67	6.86
58	Not ask permission by contributors for the wording of the acknowledgem..	5.71	4.67	6.75
59	Demand, accept or offer substantial gifts for doing a favor (C)	5.61	4.42	6.81
59	Communicate results to the general public before a peer reviewed publi..	5.61	4.46	6.76

Additional file 4.6: Ranking according to product of frequency and impact on trust

Rank	Research Misbehavior	Product Trust (mean)	Lower 95% confidence limit	Upper 95% confidence limit
1	Use published ideas or phrases of others without referencing (R)	12.08	10.66	13.50
2	Insufficiently report study flaws and limitations (R)	12.04	10.68	13.41
3	Turn a blind eye to putative breaches of research integrity by others ..	11.96	10.43	13.49
4	Insufficiently supervise or mentor junior coworkers (C)	11.81	10.55	13.07
5	Ignore basic principles of quality assurance (D)	11.76	10.40	13.11
6	Unfairly review papers, grant applications or colleagues applying for ..	11.71	10.15	13.27
7	Use unpublished ideas or phrases of others without their permission (C)	11.41	10.04	12.78
8	Demand or accept an authorship for which one does not qualify (C)	11.24	9.82	12.65
9	Inadequately handle or store data or (bio)materials (D)	11.18	9.50	12.87
10	Keep inadequate notes of the research process (D)	10.93	9.32	12.55
11	Not publish a valid 'negative' study (R)	10.92	9.24	12.60
12	Selectively cite to enhance your own findings or convictions (R)	10.75	9.42	12.09
13	Not report clearly relevant details of study methods (R)	10.70	9.28	12.11
14	Failure to disclose a relevant financial or intellectual conflict of i..	10.61	9.20	12.03
15	Be grossly unfair to your collaborators (C)	10.59	9.18	12.00
16	Handle existing conflicts of interest inadequately (C)	10.55	9.24	11.86

17	Deliberately communicate findings inaccurately in the media or during ..	10.20	8.65	11.75
18	Refuse sharing data with bona fide colleagues (C)	10.17	8.74	11.60
19	Not report all study protocol-stipulated results (R)	10.13	8.69	11.57
20	Let your convictions influence the conclusions substantially (R)	10.08	8.71	11.46
21	Selectively cite or cite your own work to improve citation metrics (R)	10.08	8.88	11.27
22	Not report replication problems (R)	10.02	8.54	11.51
23	Conceal results that contradict your earlier findings or convictions (R)	9.93	8.70	11.17
24	Selectively delete data, modify data or add fabricated data after perf..	9.86	8.52	11.20
25	Delete data before performing data analysis without disclosure (R)	9.73	8.25	11.21
26	Selectively cite to please editors, reviewers or colleagues (C)	9.61	8.17	11.05
27	Report on data-driven hypotheses without disclosure (R)	9.49	8.12	10.85
28	Re-use of previously published data without disclosure (R)	9.48	8.11	10.85
29	Add an author who doesn’t qualify for authorship (C)	9.38	8.03	10.73
30	Write no or a clearly inadequate research protocol (S)	9.38	7.95	10.80
31	Make no clear distinction between personal views and professional comm..	9.35	8.06	10.64
32	Perform data-analyses not stated in the study protocol without disclos..	9.26	7.76	10.75
33	Modify the results or conclusions of a study due to pressure of a spon..	9.12	7.62	10.62
34	Take no full responsibility for the integrity of the research project ..	9.02	7.73	10.31
35	Importantly change the research design during the study without disclo..	9.00	7.66	10.34
36	Give insufficient attention to the equipment, skills or expertise whic..	8.97	7.53	10.42
37	Omit a contributor who deserves authorship (C)	8.91	7.65	10.18
38	Propose study questions which are clearly irrelevant (S)	8.86	7.45	10.28
39	Fabricate data (D)	8.76	7.63	9.89
40	Choose a clearly inadequate research design or using evidently unsuita..	8.74	7.50	9.98
41	Not adhere to pertinent laws and regulations (D)	8.70	7.28	10.12
42	Duplicate publication without disclosure (R)	8.61	7.39	9.83
43	Refuse to respond to an allegation of a breach of research integrity (C)	8.53	7.19	9.87
44	Ignore substantial risks of the expected findings for society or envir..	8.51	7.05	9.98
45	Spread study results over more papers than needed (R)	8.45	7.33	9.58
46	Re-use parts of your own publications without referencing (R)	8.43	7.12	9.73
47	Report an incorrect downwardly rounded p-value (R)	8.27	6.65	9.89
48	Submit or resubmit a paper or grant application without consent from a..	8.26	7.18	9.34
49	Collect more data after noticing that the results are almost statistic..	8.19	6.90	9.48
50	Report an unexpected finding as having been hypothesized from the star..	8.15	6.41	9.88
51	Not acknowledge contributors who do not qualify for authorship (C)	8.11	6.82	9.39
52	Not ask permission by contributors for the wording of the acknowledgem..	7.93	6.62	9.23
53	Present grossly misleading information in a grant application (C)	7.37	6.33	8.40
54	Demand, accept or offer substantial gifts for doing a favor (C)	7.17	5.79	8.55

55	Ignore substantial safety risks of the study to participants, workers ..	7.12	5.76	8.48
56	Not share reviewers’ comments with all co-authors (C)	6.88	5.70	8.05
57	Review your own papers (C)	6.76	5.42	8.10
58	Stop data collection earlier than planned because the results are alre..	6.56	5.46	7.65
59	Failure to disclose a sponsor of the study (R)	6.43	5.30	7.56
60	Communicate results to the general public before a peer reviewed publi..	6.17	4.88	7.46

Additional file 4.7: Ranking according to product of frequency and preventability

Rank	Research Misbehavior	Product Prevent-ability (mean)	Lower 95% confidence limit	Upper 95% confidence limit
1	Insufficiently supervise or mentor junior coworkers (C)	12.96	11.57	14.36
2	Inadequately handle or store data or (bio)materials (D)	11.97	10.22	13.72
3	Use published ideas or phrases of others without referencing (R)	11.91	10.49	13.32
4	Keep inadequate notes of the research process (D)	11.89	10.37	13.40
5	Ignore basic principles of quality assurance (D)	11.40	10.11	12.68
6	Not report clearly relevant details of study methods (R)	11.24	9.81	12.67
7	Insufficiently report study flaws and limitations (R)	11.15	9.91	12.39
8	Not publish a valid 'negative' study (R)	11.08	9.52	12.63
9	Selectively cite to enhance your own findings or convictions (R)	10.96	9.68	12.24
10	Re-use parts of your own publications without referencing (R)	10.90	9.56	12.24
11	Demand or accept an authorship for which one does not qualify (C)	10.84	9.34	12.35
12	Selectively cite to please editors, reviewers or colleagues (C)	10.66	9.14	12.18
13	Not ask permission by contributors for the wording of the acknowledgem..	10.49	9.07	11.91
14	Selectively cite or cite your own work to improve citation metrics (R)	10.47	9.26	11.68
15	Spread study results over more papers than needed (R)	10.45	9.27	11.64
16	Write no or a clearly inadequate research protocol (S)	10.43	8.93	11.93
17	Add an author who doesn’t qualify for authorship (C)	10.37	8.94	11.80
18	Duplicate publication without disclosure (R)	10.30	9.05	11.55
19	Turn a blind eye to putative breaches of research integrity by others ..	10.23	8.95	11.51
20	Give insufficient attention to the equipment, skills or expertise whic..	9.92	8.45	11.40
21	Handle existing conflicts of interest inadequately (C)	9.85	8.63	11.06
22	Not report all study protocol-stipulated results (R)	9.84	8.54	11.14
23	Be grossly unfair to your collaborators (C)	9.80	8.56	11.05
24	Perform data-analyses not stated in the study protocol without disclos..	9.60	7.96	11.24
25	Submit or resubmit a paper or grant application without consent from a..	9.56	8.44	10.68

26	Use unpublished ideas or phrases of others without their permission (C)	9.51	8.21	10.81
27	Not acknowledge contributors who do not qualify for authorship (C)	9.43	8.06	10.79
28	Importantly change the research design during the study without disclo..	9.38	8.10	10.66
29	Not report replication problems (R)	9.35	8.09	10.61
30	Choose a clearly inadequate research design or using evidently unsuita..	9.27	8.01	10.54
31	Re-use of previously published data without disclosure (R)	9.25	7.98	10.52
32	Report on data-driven hypotheses without disclosure (R)	9.23	7.91	10.54
33	Omit a contributor who deserves authorship (C)	9.15	8.01	10.30
34	Failure to disclose a relevant financial or intellectual conflict of i..	9.10	7.87	10.33
35	Propose study questions which are clearly irrelevant (S)	9.06	7.46	10.65
36	Make no clear distinction between personal views and professional comm..	8.95	7.72	10.19
37	Take no full responsibility for the integrity of the research project ..	8.80	7.51	10.09
38	Report an unexpected finding as having been hypothesized from the star..	8.74	6.95	10.52
39	Unfairly review papers, grant applications or colleagues applying for ..	8.73	7.42	10.04
40	Let your convictions influence the conclusions substantially (R)	8.65	7.42	9.87
41	Refuse sharing data with bona fide colleagues (C)	8.57	7.18	9.95
42	Deliberately communicate findings inaccurately in the media or during ..	8.50	7.11	9.89
43	Delete data before performing data analysis without disclosure (R)	8.45	7.20	9.70
44	Report an incorrect downwardly rounded p-value (R)	8.42	6.80	10.05
45	Not adhere to pertinent laws and regulations (D)	8.42	7.16	9.68
46	Collect more data after noticing that the results are almost statistic..	7.96	6.74	9.18
47	Ignore substantial risks of the expected findings for society or envir..	7.94	6.61	9.28
48	Conceal results that contradict your earlier findings or convictions (R)	7.82	6.72	8.92
49	Not share reviewers’ comments with all co-authors (C)	7.71	6.53	8.88
50	Ignore substantial safety risks of the study to participants, workers ..	7.48	6.11	8.86
51	Refuse to respond to an allegation of a breach of research integrity (C)	7.42	6.19	8.66
52	Stop data collection earlier than planned because the results are alre..	7.40	6.23	8.57
53	Modify the results or conclusions of a study due to pressure of a spon..	7.22	5.94	8.50
54	Failure to disclose a sponsor of the study (R)	7.12	6.06	8.18
55	Communicate results to the general public before a peer reviewed publi..	7.06	5.80	8.32
56	Selectively delete data, modify data or add fabricated data after perf..	7.02	5.90	8.14
57	Present grossly misleading information in a grant application (C)	6.81	5.94	7.68
58	Demand, accept or offer substantial gifts for doing a favor (C)	6.42	5.24	7.59
59	Fabricate data (D)	6.40	5.46	7.34
60	Review your own papers (C)	6.32	5.06	7.58

Additional file 4.8: Ranking according to priority for RCR

Rank	Research Misbehavior	priority for RCR (mean)	Lower 95% confidence limit	Upper 95% confidence limit
1	Fabricate data (D)	4.53	4.32	4.74
2	Selectively delete data, modify data or add fabricated data after perf..	4.41	4.23	4.60
3	Turn a blind eye to putative breaches of research integrity by others ..	4.12	3.91	4.33
4	Ignore substantial safety risks of the study to participants, workers ..	4.03	3.67	4.39
5	Modify the results or conclusions of a study due to pressure of a spon..	4.02	3.73	4.32
6	Ignore basic principles of quality assurance (D)	4.02	3.81	4.24
7	Insufficiently supervise or mentor junior coworkers (C)	3.96	3.76	4.16
8	Ignore substantial risks of the expected findings for society or envir..	3.92	3.56	4.28
9	Not report all study protocol-stipulated results (R)	3.91	3.64	4.19
10	Conceal results that contradict your earlier findings or convictions (R)	3.89	3.63	4.16
11	Refuse to respond to an allegation of a breach of research integrity (C)	3.89	3.64	4.14
12	Delete data before performing data analysis without disclosure (R)	3.88	3.57	4.19
13	Failure to disclose a relevant financial or intellectual conflict of i..	3.81	3.55	4.07
14	Present grossly misleading information in a grant application (C)	3.80	3.50	4.09
15	Not adhere to pertinent laws and regulations (D)	3.78	3.48	4.08
16	Keep inadequate notes of the research process (D)	3.75	3.46	4.04
17	Handle existing conflicts of interest inadequately (C)	3.74	3.49	3.99
18	Choose a clearly inadequate research design or using evidently unsuita..	3.74	3.44	4.04
19	Take no full responsibility for the integrity of the research project ..	3.73	3.47	3.99
20	Use published ideas or phrases of others without referencing (R)	3.72	3.47	3.97
21	Inadequately handle or store data or (bio)materials (D)	3.71	3.43	4.01
22	Write no or a clearly inadequate research protocol (S)	3.69	3.42	3.96
23	Not report replication problems (R)	3.68	3.38	3.98
24	Not publish a valid 'negative' study (R)	3.66	3.33	4.00
25	Unfairly review papers, grant applications or colleagues applying for ..	3.64	3.33	3.94
25	Insufficiently report study flaws and limitations (R)	3.64	3.38	3.90
27	Not report clearly relevant details of study methods (R)	3.61	3.33	3.88
28	Let your convictions influence the conclusions substantially (R)	3.59	3.31	3.86
29	Report an incorrect downwardly rounded p-value (R)	3.53	3.25	3.81
30	Demand or accept an authorship for which one does not qualify (C)	3.53	3.25	3.81
31	Use unpublished ideas or phrases of others without their permission (C)	3.50	3.22	3.78
32	Be grossly unfair to your collaborators (C)	3.49	3.25	3.73
33	Give insufficient attention to the equipment, skills or expertise whic..	3.47	3.22	3.73
34	Deliberately communicate findings inaccurately in the media or during ..	3.46	3.18	3.75
35	Report on data-driven hypotheses without disclosure (R)	3.34	3.03	3.65
36	Importantly change the research design during the study without disclo..	3.34	3.07	3.61
37	Demand, accept or offer substantial gifts for doing a favor (C)	3.33	3.00	3.66
37	Re-use of previously published data without disclosure (R)	3.33	3.00	3.66
39	Failure to disclose a sponsor of the study (R)	3.30	3.01	3.59
40	Perform data-analyses not stated in the study protocol without disclos..	3.28	2.97	3.59
41	Review your own papers (C)	3.26	2.80	3.72
42	Selectively cite to enhance your own findings or convictions (R)	3.25	2.98	3.53
43	Duplicate publication without disclosure (R)	3.22	2.91	3.53
44	Refuse sharing data with bona fide colleagues (C)	3.19	2.87	3.51
45	Omit a contributor who deserves authorship (C)	3.15	2.89	3.41
46	Add an author who doesn’t qualify for authorship (C)	3.15	2.85	3.44
47	Submit or resubmit a paper or grant application without consent from a..	3.13	2.87	3.40
48	Not share reviewers’ comments with all co-authors (C)	3.13	2.86	3.39
49	Make no clear distinction between personal views and professional comm..	3.12	2.90	3.33
50	Selectively cite to please editors, reviewers or colleagues (C)	3.07	2.73	3.41
51	Selectively cite or cite your own work to improve citation metrics (R)	3.06	2.83	3.28
52	Report an unexpected finding as having been hypothesized from the star..	3.06	2.66	3.46
53	Stop data collection earlier than planned because the results are alre..	3.03	2.68	3.37
54	Collect more data after noticing that the results are almost statistic..	3.00	2.68	3.32
54	Spread study results over more papers than needed (R)	3.00	2.75	3.25
56	Propose study questions which are clearly irrelevant (S)	2.95	2.60	3.29
57	Re-use parts of your own publications without referencing (R)	2.94	2.63	3.25
58	Not acknowledge contributors who do not qualify for authorship (C)	2.87	2.55	3.19
59	Communicate results to the general public before a peer reviewed publi..	2.67	2.28	3.06
60	Not ask permission by contributors for the wording of the acknowledgem..	2.47	2.19	2.76
