# Supplementary material for: Ranking major and minor research misbehaviors: results from a survey among participants of four World Conferences on Research Integrity
Source: Res Integr Peer Rev. 2016 Nov 21;1:17. doi: 10.1186/s41073-016-0024-5 (PMC5803629; doi:10.1186/s41073-016-0024-5)
Supplement: Supplementary file 5 — Results of the survey. (PDF 79 kb) [file 41073_2016_24_MOESM5_ESM.pdf]

Additional file 5: Results of the survey

Research Misbehavior *	No of respondents per item (n)	Frequency		Impact on validity		Impact on trust		Preventability		Priority		Best Mode of prevention strategy data (in %)							
		Mean +/- 95% CI	# of resp.	Mean +/- 95% CI	# of resp.	Mean +/- 95% CI	# of Resp.	Mean +/- 95% CI	# of resp.	Mean +/- 95% CI	# of resp.	Education	Code/guideline	Infrastructure	Audits	Incentives	intervention in culture	Other	# of respondents
1.	55	2.71<br>2.41-<br>3.02	49	3.53<br>3.24<br>3.82	49	3.92<br>3.65<br>4.19	49	3.41<br>3.17<br>3.64	49	3.81<br>3.55<br>4.07	48	8	48	0	22	8	4	10	50
2.	46	2.73<br>2.39<br>3.07	37	3.66<br>3.33<br>3.99	38	3.50<br>3.21<br>3.79	38	3.71<br>3.41<br>4.01	38	3.69<br>3.42<br>3.96	39	50	13	5	11	0	8	13	38
3.	53	2.70<br>2.43<br>2.98	37	3.44<br>3.13<br>3.76	36	3.38<br>3.02<br>3.74	37	3.49<br>3.17<br>3.81	37	3.34<br>3.07<br>3.61	38	21	16	0	29	5	18	11	38
4.	53	3.02<br>2.70<br>3.35	46	3.61<br>3.32<br>3.90	46	3.59<br>3.32<br>3.85	46	3.67<br>3.42<br>3.93	46	3.61<br>3.33<br>3.88	46	35	24	2	9	4	20	7	46
5.	55	1.88<br>1.65<br>2.11	50	4.63<br>4.43<br>4.84	49	4.70<br>4.51<br>4.89	50	3.34<br>3.05<br>3.63	50	4.53<br>4.32<br>4.74	51	12	6	2	27	10	25	19	52
6.	49	3.11<br>2.71<br>3.50	38	3.29<br>2.91<br>3.67	38	3.58<br>3.29<br>3.87	38	3.79<br>3.50<br>4.08	38	3.71<br>3.42<br>4.01	38	15	15	23	23	0	15	8	39
7.	49	2.92<br>2.63<br>3.21	49	2.96<br>2.65<br>3.27	49	3.90<br>3.63<br>4.16	49	3.14<br>2.82<br>3.46	49	3.50<br>3.22<br>3.78	50	41	16	0	12	2	16	12	49
8.	53	2.74<br>2.43<br>3.06	47	2.96<br>2.62<br>3.29	47	3.55<br>3.23<br>3.87	47	3.07<br>2.71<br>3.42	46	3.19<br>2.87<br>3.51	47	26	6	2	4	21	30	11	47
9.	52	3.83<br>2.50<br>3.15	35	3.34<br>2.95<br>3.74	35	3.26<br>2.95<br>3.56	35	3.14<br>2.85<br>3.43	35	3.34<br>3.03<br>3.65	35	47	17	0	6	0	25	6	36
10.	53	3.46<br>3.18<br>3.74	52	3.63<br>3.39<br>3.86	51	3.40<br>3.17<br>3.64	52	3.75<br>3.49<br>4.01	52	3.96<br>3.76<br>4.16	50	21	13	10	4	19	19	13	52
11.	52	3.00<br>2.64<br>3.36	35	3.37<br>2.99<br>3.76	35	3.09<br>2.75<br>3.42	35	3.14<br>2.75<br>3.54	35	3.28<br>2.97<br>3.59	36	24	27	3	19	5	16	5	37
12.	52	3.39<br>3.09<br>3.70	38	3.27<br>2.82<br>3.72	37	3.16<br>2.76<br>3.56	38	3.24<br>2.89<br>3.59	38	3.66<br>3.32<br>4.00	38	5	10	7	7	25	20	25	40
13.	50	3.29<br>2.95<br>3.63	45	2.59<br>2.28<br>2.90	44	2.86<br>2.54<br>3.19	44	3.11<br>2.78<br>3.45	44	3.07<br>2.73<br>3.41	42	43	20	0	2	9	15	11	46

14.	47	3.07<br>2.74<br>3.41	41	1.88<br>1.61<br>2.14	41	2.59<br>2.27<br>2.90	41	3.39<br>3.12<br>3.66	41	2.47<br>2.19<br>2.76	40	44	27	2	0	12	15	0	41
15.	48	2.76<br>2.42<br>3.09	49	3.57<br>3.28<br>3.87	47	4.06<br>3.79<br>4.34	48	3.02<br>2.72<br>3.32	48	3.64<br>3.33<br>3.94	47	6	28	4	23	4	26	9	47
16.	56	3.23<br>2.95<br>3.52	47	3.77<br>3.53<br>4.01	47	3.68<br>3.41<br>3.95	47	3.40<br>3.17<br>3.64	47	3.64<br>3.38<br>3.90	47	44	10	0	6	2	27	10	48
17.	51	2.83<br>2.44<br>3.23	36	2.97<br>2.61<br>3.33	37	2.97<br>2.69<br>3.25	37	3.06<br>2.69<br>3.42	36	2.95<br>2.60<br>3.29	37	51	8	0	0	14	16	11	37
18.	50	2.78<br>2.46<br>3.09	45	2.91<br>2.57<br>3.25	46	3.31<br>2.99<br>3.63	45	3.38<br>3.13<br>3.62	45	3.33<br>3.00<br>3.66	45	30	24	4	11	2	22	7	46
19.	51	2.75<br>2.42<br>3.08	40	3.47<br>3.10<br>3.85	40	3.75<br>3.42<br>4.08	40	3.13<br>2.79<br>3.46	40	3.46<br>3.18<br>3.75	41	18	20	0	5	13	28	18	40
20.	49	3.35<br>3.02<br>3.69	51	2.29<br>1.98<br>2.61	51	3.20<br>2.92<br>3.47	51	3.24<br>2.93<br>3.54	51	3.53<br>3.25<br>3.81	51	10	37	0	6	14	31	2	51
21.	56	3.27<br>2.99<br>3.55	51	2.63<br>2.39<br>2.88	52	2.98<br>2.72<br>3.24	52	3.13<br>2.88<br>3.39	52	3.06<br>2.83<br>3.28	52	26	22	6	6	16	16	8	50
22.	58	1.91<br>1.60<br>2.22	33	3.15<br>2.69<br>3.60	34	3.74<br>3.37<br>4.10	34	3.91<br>3.58<br>4.25	34	4.03<br>3.67<br>4.39	34	18	12	15	18	0	21	18	34
23.	60	3.13<br>2.81<br>3.44	48	3.78<br>3.52<br>4.04	50	3.90<br>3.60<br>4.20	50	3.26<br>3.01<br>3.51	50	4.12<br>3.91<br>4.33	50	14	14	0	8	8	42	14	50
24.	55	3.16<br>2.80<br>3.51	45	3.82<br>3.56<br>4.09	45	3.47<br>3.14<br>3.79	45	3.70<br>3.47<br>3.94	44	3.75<br>3.46<br>4.04	44	11	43	9	9	13	9	7	46
25.	57	2.48<br>2.15<br>2.80	42	4.02<br>3.71<br>4.34	42	3.95<br>3.65<br>4.26	42	3.29<br>3.03<br>3.56	41	3.88<br>3.57<br>4.19	42	23	9	7	14	2	28	16	43
26.	56	2.30<br>1.92<br>2.69	33	3.50<br>3.16<br>3.84	34	3.59<br>3.23<br>3.95	34	3.62<br>3.26<br>3.98	34	3.53<br>3.25<br>3.81	32	53	9	0	15	6	0	18	34
27.	54	2.12<br>1.84<br>2.41	41	2.93<br>2.63<br>3.23	41	3.34<br>3.02<br>3.67	41	3.63<br>3.37<br>3.90	41	3.13<br>2.86<br>3.39	40	24	22	12	7	12	7	15	41
28.	54	2.88<br>2.43<br>3.33	34	2.83<br>2.41<br>3.25	35	2.94<br>2.56<br>3.33	35	3.06<br>2.66<br>3.45	35	3.06<br>2.66<br>3.46	35	44	6	0	17	3	14	17	36
29.	56	2.55<br>2.19<br>2.91	40	3.70<br>3.47<br>3.93	40	3.44<br>3.14<br>3.73	39	3.77<br>3.54<br>4.00	39	3.47<br>3.22<br>3.73	40	33	18	20	8	0	8	15	40
30.	56	2.74<br>2.44<br>3.05	43	3.73<br>3.39<br>4.06	44	3.59<br>3.23<br>3.95	44	3.41<br>3.15<br>3.67	44	3.68<br>3.38<br>3.98	44	16	14	11	14	5	32	9	44
31.	58	3.16<br>2.89<br>3.44	55	2.38<br>2.11<br>2.64	56	2.63<br>2.35<br>2.90	56	3.29<br>3.05<br>3.52	56	3.00<br>2.75<br>3.25	55	23	16	2	5	18	21	16	57
32.	56	2.10<br>1.78<br>2.41	42	4.36<br>4.13<br>4.59	42	4.40<br>4.15<br>4.66	42	3.54<br>3.25<br>3.83	41	4.02<br>3.73<br>4.32	42	12	17	5	12	17	17	21	42

33.	59	2.81<br>2.53<br>3.09	53	3.55<br>3.26<br>3.83	53	3.68<br>3.39<br>3.97	53	3.45<br>3.19<br>3.71	53	3.74<br>3.49<br>3.99	50	19	30	4	13	6	21	8	53
34.	56	3.00<br>2.67<br>3.33	43	3.14<br>2.84<br>3.45	42	3.09<br>2.83<br>3.36	43	3.00<br>2.75<br>3.25	43	3.12<br>2.90<br>3.33	43	51	7	0	5	2	28	7	43
35.	56	3.11<br>2.82<br>3.41	53	2.94<br>2.63<br>3.26	54	3.81<br>3.54<br>4.09	54	3.81<br>3.55<br>4.08	54	3.72<br>3.47<br>3.97	53	37	15	2	17	2	15	13	54
36.	59	2.52<br>2.24<br>2.80	44	4.18<br>3.93<br>4.42	45	3.43<br>3.12<br>3.74	44	3.71<br>3.43<br>3.99	45	3.74<br>3.44<br>4.04	46	48	2	7	9	4	11	20	46
37.	55	1.68<br>1.41<br>1.95	25	4.00<br>3.57<br>4.43	26	4.08<br>3.63<br>4.52	26	3.88<br>3.47<br>4.30	26	3.26<br>2.80<br>3.72	27	7	26	11	19	0	19	19	27
38.	55	2.39<br>2.13<br>2.65	36	3.16<br>2.79<br>3.53	37	2.86<br>2.53<br>3.20	37	3.17<br>2.82<br>3.51	36	3.03<br>2.68<br>3.37	36	38	14	5	11	3	14	16	374
39.	58	2.86<br>2.55<br>3.17	51	2.74<br>2.44<br>3.04	50	3.63<br>3.33<br>3.92	51	3.37<br>3.14<br>3.61	51	3.49<br>3.25<br>3.73	51	12	20	2	4	12	37	14	51
40.	55	2.68<br>2.39<br>2.97	47	2.30<br>2.05<br>2.55	47	3.30<br>2.98<br>3.61	47	3.33<br>3.09<br>3.56	46	3.15<br>2.89<br>3.41	47	11	38	4	2	13	23	9	47
41.	60	2.77<br>2.45<br>3.08	47	3.13<br>2.81<br>3.45	47	2.94<br>2.61<br>3.26	47	2.83<br>2.52<br>3.14	47	3.00<br>2.68<br>3.32	45	39	20	4	7	4	22	4	46
42.	52	2.54<br>2.28<br>2.81	46	4.04<br>3.78<br>4.31	47	3.94<br>3.67<br>4.20	48	3.11<br>2.82<br>3.40	45	3.89<br>3.63<br>4.16	47	21	10	4	21	10	21	13	48
43.	54	2.02<br>1.76<br>2.29	42	3.09<br>2.75<br>3.43	44	3.27<br>2.91<br>3.64	44	3.61<br>3.38<br>3.85	44	3.30<br>3.01<br>3.59	43	9	44	2	26	5	9	5	43
44.	54	2.22<br>1.94<br>2.50	50	4.37<br>4.11<br>4.62	52	4.48<br>4.28<br>4.69	52	3.22<br>2.92<br>3.51	51	4.41<br>4.22<br>4.60	49	31	8	2	29	4	16	10	51
45.	57	2.17<br>1.87<br>2.48	35	3.25<br>2.76<br>3.74	36	3.92<br>3.53<br>4.30	36	3.57<br>3.22<br>3.92	35	3.92<br>3.56<br>4.28	37	41	19	5	3	8	19	5	37
46.	56	2.91<br>2.61<br>3.22	46	3.61<br>3.30<br>3.92	46	3.57<br>3.25<br>3.90	47	3.37<br>3.09<br>3.64	46	3.91<br>3.64<br>4.19	46	22	24	4	15	4	17	13	46
47.	59	2.98<br>2.69<br>3.27	49	4.00<br>3.79<br>4.21	49	3.94<br>3.69<br>4.18	49	3.83<br>3.61<br>4.05	48	4.02<br>3.81<br>4.24	48	38	15	4	13	13	17	2	48
48.	56	3.53<br>3.26<br>3.80	53	3.30<br>3.02<br>3.57	54	3.15<br>2.86<br>3.44	54	3.17<br>2.90<br>3.43	54	3.25<br>2.98<br>3.53	51	44	11	4	4	4	22	11	54
49.	52	2.94<br>2.63<br>3.25	48	3.74<br>3.47<br>4.01	50	3.44<br>3.15<br>3.73	50	2.94<br>2.66<br>3.22	50	3.59<br>3.31<br>3.86	51	57	0	0	12	4	20	8	51
50.	52	2.74<br>2.47<br>3.01	50	2.65<br>2.28<br>3.02	51	3.20<br>2.89<br>3.51	51	3.79<br>3.53<br>4.04	52	3.22<br>2.91<br>3.53	50	21	21	8	19	13	13	6	53
51.	57	2.13<br>1.84<br>2.43	45	3.84<br>3.52<br>4.17	45	4.00<br>3.71<br>4.29	46	3.52<br>3.21<br>3.83	46	3.89<br>3.64<br>4.14	46	15	15	13	11	4	28	15	47

52.	56	3.04<br>2.72<br>3.36	50	2.37<br>2.10<br>2.64	54	2.83<br>2.51<br>3.14	52	3.55<br>3.31<br>3.79	53	2.94<br>2.63<br>3.25	52	32	15	6	13	4	15	15	53
53.	51	2.35<br>2.04<br>2.67	37	2.46<br>2.10<br>2.82	37	2.76<br>2.35<br>3.16	37	3.19<br>2.87<br>3.51	36	2.67<br>2.28<br>3.06	36	25	42	3	3	6	17	6	36
54.	61	2.76<br>2.46<br>3.07	46	3.00<br>2.68<br>3.32	46	3.30<br>3.01<br>3.60	46	3.24<br>2.94<br>3.54	46	3.73<br>3.47<br>3.99	45	27	21	4	6	10	27	4	48
55.	53	2.06<br>1.74<br>2.37	36	2.92<br>2.54<br>3.29	37	3.78<br>3.43<br>4.13	36	3.19<br>2.89<br>3.49	37	3.33<br>3.00<br>3.66	36	11	32	3	14	3	27	11	37
56.	53	3.20<br>2.90<br>3.50	55	2.07<br>1.77<br>2.37	57	2.98<br>2.67<br>3.30	57	3.21<br>2.88<br>3.54	56	3.15<br>2.85<br>3.44	55	11	39	0	4	2	37	9	57
57.	48	2.07<br>1.86<br>2.29	42	3.34<br>2.99<br>3.69	44	3.70<br>3.38<br>4.01	43	3.34<br>3.10<br>3.58	44	3.80<br>3.50<br>4.09	44	16	14	5	39	7	16	5	44
58.	54	2.35<br>2.03<br>2.67	43	3.51<br>3.17<br>3.86	45	3.80<br>3.48<br>4.12	45	3.49<br>3.23<br>3.74	45	3.78<br>3.48<br>4.08	45	29	20	2	24	4	13	7	45
59.	54	2.83<br>2.54<br>3.12	47	2.00<br>1.68<br>2.32	48	2.81<br>2.46<br>3.16	48	3.27<br>2.93<br>3.61	48	2.87<br>2.55<br>3.19	47	25	48	0	6	4	15	2	48
60.	54	2.52<br>2.29<br>2.75	50	2.47<br>2.14<br>2.80	53	3.19<br>2.88<br>3.50	53	3.73<br>3.45<br>4.01	52	3.13<br>2.87<br>3.40	53	19	36	8	6	2	19	11	53

* See appendix of Additional file 3 for the specific research misbehaviors corresponding to these numbers.
