# Supplementary material for: Ranking major and minor research misbehaviors: results from a survey among participants of four World Conferences on Research Integrity
Source: Res Integr Peer Rev. 2016 Nov 21;1:17. doi: 10.1186/s41073-016-0024-5 (PMC5803629; doi:10.1186/s41073-016-0024-5)
Supplement: Supplementary file 6 — Top 5 rankings for disciplinary fields. (PDF 111 kb) [file 41073_2016_24_MOESM6_ESM.pdf]

Additional file 6: Top 5 rankings according to frequency, impact on truth and trust, and preventability – stratified for disciplinary field[1]

Additional file 6.1. Top 5 rankings according to frequency, impact on truth and trust, and preventability - biomedical sciences (No of respondents per item ranging from 24-29)

Rank number	Frequency (1-5)	mean score (95% confidence interval)
1	Selectively cite or cite your own work to improve citation metrics (R)	**3.70** (3.24 - 4.15)
2	Demand or accept an authorship for which one does not qualify (C)	**3.68** (3.29 - 4.07)
2	Selectively cite to enhance your own findings or convictions (R)	**3.54** (3.14 - 3.93)
4	Add an author who doesn't qualify for authorship (C)	**3.54** (3.09 - 3.98)
5	Turn a blind eye to putative breaches of research integrity by others (C)	**3.50** (3.00 - 4.00)

Rank number	Impact on truth (1-5)	mean score (95% confidence interval)
1	Fabricate data (D)	**4.78** (4.56 – 5.00)
2	Modify the results or conclusions of a study due to pressure of a sponsor (R)	**4.68** (4.44 - 4.92)
3	Selectively delete data, modify data or add fabricated data after performing initial data-analyses (R)	**4.46** (4.14 - 4.79)
4	Choose a clearly inadequate research design or using evidently unsuitable measurement instruments (S)	**4.26** (4.01 - 4.51)
5	Conceal results that contradict your earlier findings or convictions (R)	**4.18** (3.89 - 4.46)

Rank number	Impact on trust (1-5)	mean score (95% CI)
1	Fabricate data (D)	**4.78** (4.56 - 5.00)
2	Selectively delete data, modify data or add fabricated data after performing initial data-analyses (R)	**4.54** (4.26 - 4.81)

[1] Data for the stratum Humanities are not presented due to the small number of respondents per item (range 1– 8).

3	Modify the results or conclusions of a study due to pressure of a sponsor (R)	4.41 (4.05 - 4.77)
4	Refuse to respond to an allegation of a breach of research integrity (C)	4.14 (3.72 - 4.55)
5	Unfairly review papers, grant applications or colleagues applying for promotion (C)	4.07 (3.75 - 4.39)

Rank number	Preventability (1-5)	mean score (95% confidence interval)
1	Insufficiently supervise or mentor junior coworkers (C)	3.97 (3.67 - 4.27)
2	Duplicate publication without disclosure (R)	3.92 (3.64 - 4.21)
3	Submit or resubmit a paper or grant application without consent from all authors (C)	3.92 (3.56 - 4.28)
4	Write no or a clearly inadequate research protocol (S)	3.92 (3.58 - 4.26)
5	Inadequately handle or store data or (bio)materials (D)	3.92 (3.56 - 4.28)

Rank number	Product of frequency and impact on truth (1-25)	mean score (95% confidence interval)
1	Turn a blind eye to putative breaches of research integrity by others (C)	12.59 (11.29 - 13.89)
2	Keep inadequate notes of the research process (D)	12.32 (10.99 - 13.65)
3	Not publish a valid 'negative' study (R)	12.18 (10.57 - 13.78)
4	Ignore basic principles of quality assurance (D)	12.12 (10.69 - 13.56)
5	Not report replication problems (R)	12.04 (10.72 - 13.36)

Rank number	Product of frequency and impact on trust (1-25)	mean score (95% CI)
1	Turn a blind eye to putative breaches of research integrity by others (C)	13.90 (11.26 - 16.54)
2	Demand or accept an authorship for which one does not qualify (C)	12.68 (10.86 - 14.50)

3	Use unpublished ideas or phrases of others without their permission (C)	12.32 (10.63 - 14.02)
4	Ignore basic principles of quality assurance (D)	12.17 (10.18 - 14.17)
5	Not publish a valid 'negative' study (R)	12.12 (10.19 - 14.05)

Rank number	Product of frequency and preventability (1-25)	mean score (95% confidence interval)
1	Insufficiently supervise or mentor junior coworkers (C)	13.66 (11.74 – 15.57)
2	Inadequately handle or store data or (bio)materials (D)	13.36 (11.05 - 15.67)
3	Demand or accept an authorship for which one does not qualify (C)	13.07 (11.02 - 15.13)
4	Keep inadequate notes of the research process (D)	12.76 (10.57 - 14.95)
5	Not publish a valid 'negative' study (R)	12.48 (10.69 – 14.27)

Additional file 6.2: Top 5 rankings according to frequency, impact on truth and trust, and preventability - social sciences (No of respondents per item ranging from 5-12)

Rank number	Frequency (1-5)	mean score (95% confidence interval)
1	Insufficiently supervise or mentor junior coworkers (C)	3.71 (3.15 - 4.27)
2	Not report clearly relevant details of study methods (R)	3.57 (2.73 - 4.41)
2	Insufficiently report study flaws and limitations (R)	3.50 (2.83 - 4.17)
4	Not ask permission by contributors for the wording of the acknowledgement (C)	3.50 (2.66 – 4.34)
5	Spread study results over more papers than needed (R)	3.50 (2.76 - 4.24)

Rank number	Impact on truth (1-5)	mean score (95% confidence interval)
1	Review your own papers (C)	5.00 (5.00 – 5.00)
2	Fabricate data (D)	4.57 (3.99 - 5.15)
3	Modify the results or conclusions of a study due to pressure of a sponsor (R)	4.40 (3.92 - 4.88)

4	Selectively delete data, modify data or add fabricated data after performing initial data-analyses (R)	4.36 (3.65 - 5.08)
5	Choose a clearly inadequate research design or using evidently unsuitable measurement instruments (S)	4.29 (3.73 - 4.85)

Rank number	Impact on trust (1-5)	mean score (95% CI)
1	Review your own papers (C)	5.00 (5.00 - 5.00)
2	Fabricate data (D)	4.86 (4.58 – 5.14)
3	Modify the results or conclusions of a study due to pressure of a sponsor (R)	4.80 (4.41 – 5.19)
4	Demand, accept or offer substantial gifts for doing a favor (C)	4.57 (4.18 - 4.97)
5	Selectively delete data, modify data or add fabricated data after performing initial data-analyses (R)	4.55 (3.99 – 5.10)

Rank number	Preventability (1-5)	mean score (95% confidence interval)
1	Review your own papers (C)	5.00 (5.00 – 5.00)
2	Ignore substantial safety risks of the study to participants, workers or environment (S)	4.60 (3.82 – 5.38)
3	Ignore basic principles of quality assurance (D)	4.10 (3.64 – 4.56)
4	Duplicate publication without disclosure (R)	4.08 (3.71 - 4.46)
5	Give insufficient attention to the equipment, skills or expertise which are essential to perform the study (S)	3.83 (3.23 - 4.44)

Rank number	Product of frequency and impact on truth (1-25)	mean score (95% confidence interval)
1	Insufficiently supervise or mentor junior coworkers (C)	14.50 (11.39 – 17.61)
2	Insufficiently report study flaws and limitations (R)	13.83 (10.51 – 17.16)
3	Write no or a clearly inadequate research protocol (S)	12.60 (7.42 – 17.78)
4	Choose a clearly inadequate research design or using evidently unsuitable measurement instruments (S)	12.29 (10.28 – 14.29)

5	Not report clearly relevant details of study methods (R)	12.00 (8.12 – 15.88)

Rank number	Product of frequency and impact on trust (1-25)	mean score (95% CI)
1	Ignore basic principles of quality assurance (D)	12.40 (9.51 - 15.29)
2	Delete data before performing data analysis without disclosure (R)	12.00 (7.46 - 16.54)
3	Insufficiently report study flaws and limitations (R)	11.50 (7.59 - 15.41)
4	Insufficiently supervise or mentor junior coworkers (C)	11.43 (8.20 - 14.66)
5	Failure to disclose a relevant financial or intellectual conflict of interest (R)	11.38 (7.74 - 15.01)

Rank number	Product of frequency and preventability (1-25)	mean score (95% confidence interval)
1	Insufficiently supervise or mentor junior coworkers (C)	12.43 (9.12 – 15.74)
2	Not report clearly relevant details of study methods (R)	12.14 (8.58 - 15.71)
3	Selectively cite to enhance your own findings or convictions (R)	11.64 (8.94 - 14.34)
4	Insufficiently report study flaws and limitations (R)	11.50 (8.50 - 14.50)
5	Ignore basic principles of quality assurance (D)	11.50 (8.91 – 14.09)

Additional file 6.3: Top 5 rankings according to frequency, impact on truth and trust, and preventability - natural sciences (No of respondents per item ranging from 4-9)

Rank number	Frequency (1-5)	mean score (95% confidence interval)
1	Selectively cite to please editors, reviewers or colleagues (C)	3.75 (3.03 - 4.47)
2	Deliberately communicate findings inaccurately in the media or during presentation (R)	3.50 (2.83 - 4.17)
2	Unfairly review papers, grant applications or colleagues applying for promotion (C)	3.33 (2.68 - 3.99)
4	Insufficiently supervise or mentor junior coworkers (C)	3.33 (2.68 - 3.99)

5	Selectively cite to enhance your own findings or convictions (R)	3.25 (2.31 - 4.19)

Rank number	Impact on truth (1-5)	mean score (95% confidence interval)
1	Report an incorrect downwardly rounded p-value (R)	4.33 (3.68 – 4.99)
2	Conceal results that contradict your earlier findings or convictions (R)	4.33 (3.03 – 5.64)
3	Selectively delete data, modify data or add fabricated data after performing initial data-analyses (R)	4.25 (3.31 - 5.19)
4	Fabricate data (D)	4.22 (3.51 - 4.94)
5	Delete data before performing data analysis without disclosure (R)	4.20 (3.06 - 5.34)

Rank number	Impact on trust (1-5)	mean score (95% CI)
1	Report an incorrect downwardly rounded p-value (R)	4.67 (4.01 - 5.32)
2	Modify the results or conclusions of a study due to pressure of a sponsor (R)	4.57 (4.18 - 4.97)
3	Selectively delete data, modify data or add fabricated data after performing analysis (R)	4.50 (3.93 - 5.07)
4	Review your own papers (C)	4.40 (3.62 - 5.18)
5	Turn a blind eye to putative breaches of research integrity by others (C)	4.29 (3.58 - 4.99)

Rank number	Preventability (1-5)	mean score (95% confidence interval)
1	Ignore substantial safety risks of the study to participants, worker or environment (S)	4.17 (3.38 - 4.95)
2	Not share reviewers' comments with all co-authors (C)	4.14 (3.86 - 4.42)
3	Use published ideas or phrases of others without referencing (R)	4.13 (3.68 - 4.57)
4	Handle existing conflicts of interest inadequately (C)	4.13 (3.68 - 4.57)
5	Keep inadequate notes of the research process (D)	4.11 (3.60 - 4.62)

Rank number	Product of frequency and impact on truth (1-25)	mean score (95% confidence interval)
1	Unfairly review papers, grant applications or colleagues applying for promotion (C)	12.67 (9.80 - 15.53)
2	Keep inadequate notes of the research process (D)	12.00 (8.28 – 15.72)
3	Not report clearly relevant details of study methods (R)	11.83 (8.27 – 15.39)
4	Communicate results to the general public before a peer review publication is available (R)	11.75 (7.74 – 15.76)
5	Turn a blind eye to putative breaches of research integrity by others (C)	11.71 (6.49 – 16.94)

Rank number	Product of frequency and impact on trust (1-25)	mean score (95% CI)
1	Unfairly review papers, grant applications or colleagues applying for promotion (C)	14.44 (11.14 – 17.75)
2	Use unpublished ideas or phrases of others without referencing (R)	13.00 (8.60 – 17.40)
3	Be grossly unfair to your collaborators (C)	12.88 (8.08 – 17.67)
4	Turn a blind eye to putative breaches of research integrity by others (C)	12.57 (7.27 – 17.88)
5	Not report clearly relevant details of study methods (R)	12.17 (8.59 - 15.74)

Rank number	Product of frequency and preventability (1-25)	mean score (95% confidence interval)
1	Use published ideas or phrases of others without referencing (R)	12.88 (8.75 – 17.00)
2	Insufficiently supervise or mentor junior coworkers (C)	12.44 (9.42 - 15.46)
3	Be grossly unfair to your collaborators (C)	12.38 (8.27 - 16.48)
4	Keep inadequate notes of the research process (D)	12.22 (8.50 - 15.94)
5	Selectively cite to please editors, reviewers or colleagues (C)	11.86 (7.66 – 16.057)
